# A Novel Group of *Moraxella catarrhalis* UspA Proteins Mediates Cellular Adhesion via CEACAMs and Vitronectin

**DOI:** 10.1371/journal.pone.0045452

**Published:** 2012-09-25

**Authors:** Darryl J. Hill, Cheryl Whittles, Mumtaz Virji

**Affiliations:** School of Cellular & Molecular Medicine, University of Bristol, Bristol, United Kingdom; University of Helsinki, Finland

## Abstract

*Moraxella catarrhalis* (Mx) is a common cause of otitis media and exacerbation of chronic obstructive pulmonary disease, an increasing worldwide problem. Surface proteins UspA1 and UspA2 of Mx bind to a number of human receptors and may function in pathogenesis. Genetic recombination events in the pathogen can generate hybrid proteins termed UspA2H. However, whether certain key functions (e.g. UspA1-specific CEACAM binding) can be exchanged between these adhesin families remains unknown. In this study, we have shown that Mx can incorporate the UspA1 CEACAM1-binding region not only into rare UspA1 proteins devoid of CEACAM-binding ability, but also into UspA2 which normally lack this capacity. Further, a screen of Mx isolates revealed the presence of novel UspA2 Variant proteins (UspA2V) in ∼14% of the CEACAM-binding population. We demonstrate that the expression of UspA2/2V with the CEACAM-binding domain enable Mx to bind both to cell surface CEACAMs and to integrins, the latter via vitronectin. Such properties of UspA2/2V have not been reported to date. The studies demonstrate that the UspA family is much more heterogeneous than previously believed and illustrate the *in vivo* potential for exchange of functional regions between UspA proteins which could convey novel adhesive functions whilst enhancing immune evasion.

## Introduction

*Moraxella catarrhalis* (Mx), a human specific acapsulate bacterium, has the capacity to cause a range of pathologies including localised infections of the upper and lower respiratory tract as well as disseminated infections such as meningitis and septicaemia in susceptible individuals [1]. However, it is most commonly associated with two pathologies: first, inflammation of the middle ear, otitis media (OM) which affects the majority of children under the age of 5 in the UK [2]; and second, exacerbation of chronic obstructive pulmonary disease (COPD) [3], which is a significant burden to human health affecting over 210 million people world wide. COPD is currently the fifth leading cause of death globally [4]. In spite of its burden to human health no vaccine is currently available to protect against Mx infection. However, a more rapid advance in this area may now be facilitated following publication of the first complete fully annotated genome of Mx [5].

A number of adhesins produced by Mx have been identified (Reviewed in [6]). Amongst the most studied adhesins of Mx are the ubiquitous surface proteins (UspA1 and UspA2), members of the trimeric autotransporter adhesin family [7]. Since the early descriptions of UspA proteins [8], [9], much work has been performed elucidating the varying functional characteristics of these proteins. Studies relating to UspA1 proteins have defined their ability to bind to a range of human epithelial cell lines including Chang [10], HEp-2 [11] and A549 cells [12]. UspA1-cellular interactions occur via members of the Carcinoembryonic antigen related cell adhesion molecule (CEACAM) subfamily [12], [13] which are also targeted by several other respiratory pathogens including *Neisseria meningitidis* (Nm) and *Haemophilus influenzae* (Hi) [14], [15]. Human CEACAM1 is widely distributed on epithelial cells of the respiratory tract [16]. Other epithelial CEACAM family members such as CEA and CEACAM6 also bind to UspA1 [13], although these interactions are less well studied. In addition to CEACAMs, cellular interactions mediated by UspA1 have been shown to involve fibronectin and subsequent engagement of fibronectin-binding integrins [17]. UspA1 proteins have also been shown to interact with laminin [18], although no cell-mediated interaction has been reported for UspA1 via laminin. The other targets of UspA1 reported are the serum enzyme inhibitor, α-anti-chymotrypsin [19], the complement factor C3 [20] and the complement regulator (C4 binding protein (C4bp) [21]). For UspA2 proteins interactions with fibronectin [11], [17], vitronectin [11] and a range of complement factors including C3 and C4bp [20], [21] have been reported. Whereas the UspA1 proteins are generally associated with an adhesive function [22], [23], UspA2 proteins are more commonly associated with a substantial level of resistance to complement-mediated killing via their interaction with C4bp and vitronectin, whilst the low levels of C4bp binding to UspA1 appear to contribute to serum resistance to a lesser extent [21], [24].

Initial studies on UspA1 and UspA2 reported that proteins of the four isolates examined shared a region of commonality of 140 amino acids with 93% identity [9]. The common region contained the epitope for the protective antibody 17C7 tentatively assigned to the presence of the NINNY motif [8], [25]; although other studies have identified that not all UspA proteins possess this epitope [26]. Further investigations on the molecular nature of UspA proteins identified a number of modules or motifs, the presence of which varies between different UspA proteins. For example, modules that mediate CEACAM-binding have only been identified within UspA1 proteins sequenced thus far; whilst the repetitive motif Ser-Ile-Glu (SIE), appeared to be restricted to UspA2 proteins [26], [27]. Our studies have previously demonstrated that CEACAMs are bound by Mx via UspA1 proteins [12], [13]. Further, the region responsible for CEACAM binding was located within amino acids 578–597 of strain MX2 [27] within the so called rD-7 region [13]. Strains that lack the region responsible for CEACAM binding i.e. 035E and TTA37 fail to bind CEACAM1 [27]. This was confirmed in other studies that showed no CEACAM1 binding by the Mx strain ATCC43617 that lacked the sequence equivalent to rD-7 [28]. As a relatively small number of UspA proteins have been sequenced to date, we examined the distribution of CEACAM1-binding ability across a range of Mx isolates. In addition, we investigated if key functions such as CEACAM binding may be transferred between UspA1/UspA2 proteins through recombination, such an event has not been recorded previously.

Here we report that the CEACAM-binding region of MX2 *uspA1* can recombine into the *uspA* genes of strain 035E conveying CEACAM binding to both UspA1 and UspA2 proteins within this strain. In addition, a screen of Mx isolates revealed the presence of a novel group of UspA proteins with UspA2-like properties in ∼14% of CEACAM1 binding strains tested. These proteins possessed the capacity to bind to CEACAMs as well as vitronectin. Additionally, and for the first time, we have demonstrated that vitronectin can mediate adhesion of Mx to human epithelial cells. The implications of these findings relating to our understanding of the UspA protein family and vaccine design are discussed.

## Results

### Transformation with a CEACAM binding uspA1 gene leads to acquisition of CEACAM binding function by *M. catarrhalis*

**Figure 1:**
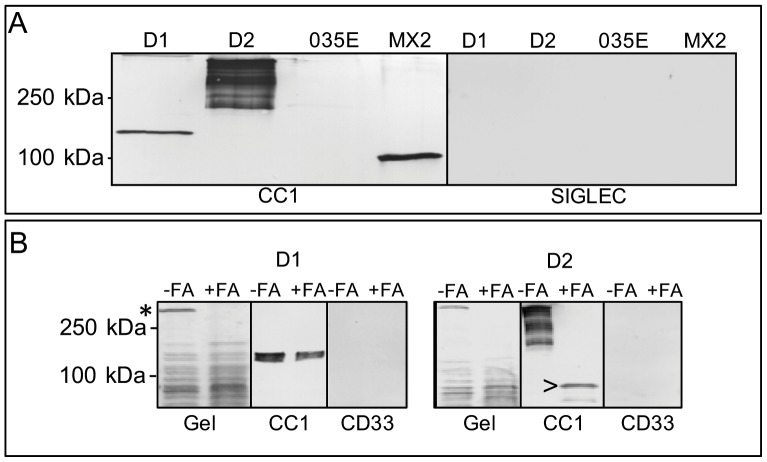
CEACAM1 binding properties of *M. catarrhalis* strain 035E and its derivatives. A) Western blot of Mx strains from right to left MX2 (used for comparison), 035E and 035E D2 and D1 overlaid with CEACAM1-Fc (CC1) or SIGLEC10-Fc (SIGLEC) as described in the methods. As expected, MX2 UspA1 bound to CEACAM1-Fc. No CEACAM binding protein was observed for 035E while its transformants D1 and D2 both bound to CEACAM1. Of these proteins, D2 migrated at a much higher molecular weight than expected for UspA1. B) SDS-PAGE gels stained with Coomassie (Gel) and corresponding Western blots (CC1 and CD33) overlaid with CEACAM1-Fc and CD33-Fc respectively. Bacterial lysates of 035E D1 and D2 were preincubated without (−FA) or with 70% formic acid (+FA) and then heated at 100°C for 5 min. In the case of D1, a high molecular weight band (*) is no longer seen in the gel after formic acid treatment and one prominent CEACAM-binding band was observed in the Western blot with or without prior formic acid treatment. Thus heat alone (−FA) appears to be sufficient to induce a level of dissociation of the protein and so affect the migration of the protein, whereas formic acid treatment results in its complete dissociation (+FA). In the case of D2, formic acid treatment was required for the dissociation of the D2 high molecular weight band in the gel and correlated with the appearance of a lower molecular weight CEACAM binding band (>). Note the laddering effect on the D2 CEACAM-binding blot in the absence of formic acid is characteristic of some oligomeric coiled coil adhesins. Whilst regions of interest are presented here, full gel and blot images are shown in Fig. S1.

Repetitive sequences present within *uspA* genes of Mx suggests inter- and intra- genomic recombination events could occur between *uspA* genes leading to the expression of novel UspA proteins possessing altered functional properties. In order to test this hypothesis, the *uspA1* gene from strain MX2 (which possesses the rD-7 region shown to mediate binding to CEACAMs; [13]) was used to transform 035E (the UspA1 of which lacks the majority of the rD-7 region; [27]). Following transformation, bacteria were grown on HBHI agar and colony blotted. Blots were overlaid with CEACAM1-Fc and the colonies that bound CEACAM1-Fc were selected for further analysis. When whole bacterial cell lysates of CEACAM1 binding colonies were subjected to SDS-PAGE and Western blots of the gels overlaid with CEACAM1-Fc, no CEACAM1 binding protein was observed for the parental strain 035E as reported previously [27], [28]. However, of the CEACAM-binding 035E variants, two different CEACAM1 binding variants were apparent (Fig. 1A, Fig. S1). One derivative (termed 035E D1) possessed a CEACAM binding protein at ∼120 kDa, the second (termed 035E D2) possessed a higher molecular weight CEACAM1 binding profile and several bands could be observed >250 kDa (035E D2 Fig. 1A). These data suggested that the motif encoding the CEACAM1 binding region of MX2 *uspA1* had recombined within the genome of 035E leading the functional expression of the CEACAM-binding motif. In addition, it would appear that two independent recombination events have occurred within the strain 035E. This is the first time the functional domain responsible for CEACAM binding has been shown to be transferred between strains of *M. catarrhalis*.

### Nature of the novel CEACAM binding proteins within 035E

We have previously shown heating to be sufficient to allow the oligomeric UspA1 proteins carrying the CEACAM binding motifs to dissociate into monomers [12], [13]; a property well recognised for the UspA1 proteins [11]. Accordingly, the CEACAM1-binding protein of D1 of the strain 035E was heat modifiable (Fig. 1A) and its monomers migrated with an apparent molecular weight of ∼120 kDa (Fig. 1B). However, D2 behaved in a different manner as it did not dissociate into monomers on heating and migrated as a high molecular weight oligomer, a behaviour typically associated with UspA2 (Fig. 1A) [11].

On the other hand, formic acid treatment of Mx can be used to dissociate UspA proteins into monomers [29]. In order to confirm the UspA2-like nature of the D2 CEACAM binding phenotype, bacterial lysates were solubilised with 70% formic acid prior to electrophoresis. This treatment resulted in dissociation of D2 into monomers that migrated at approximately 80 kDa and bound to CEACAM1 (Fig. 1B). Based on the treatment required for the dissociation of D1 and D2 and their relative monomer size (UspA2 proteins are typically smaller than UspA1, [25]) the data suggest integration of the CEACAM binding domain from MX2 *uspA1* into *uspA1*gene for D1 of 035E, and unexpectedly, the *uspA2* gene of D2.

### Sequencing the *uspA* variant genes

**Figure 2:**
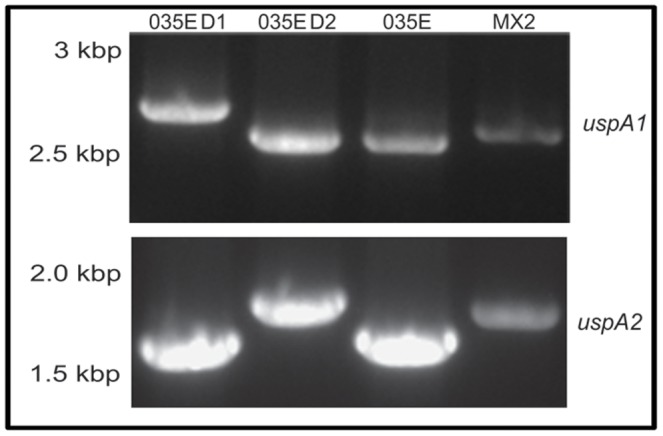
Identification of *uspA* genes from *M. catarrhalis* CEACAM-binding variants. PCR of Mx D1, D2, 035E and MX2 for *uspa1* (upper panel) and *uspA2* (lower panel). Compared to 035E, *uspA1* showed an increase in size in D1, whilst *uspA2* of D2 was larger than the parental 035E. Note *uspA1* of D1was larger than both 035E and MX2 genes. Data are representative of PCR products obtained on several occasions.

In order to confirm if the CEACAM binding motif had indeed integrated into both UspA1 and UspA2 of strain 035E, and if so the position of integration, *uspA1*, and *uspA2* genes were amplified from MX2, 035E, D1 and D2 by PCR using conserved primer pairs (Fig. 2). For the Mx strain MX2 gene products of ∼2.6 kb and 1.8 kb were obtained which corresponded to *uspA1* and *uspA2* respectively and for 035E, gene products of ∼2.5 kb and 1.6 kb were obtained for *uspA1* and *uspA2* respectively. All four gene products were of the expected sizes for these strains based on the primer positions and available sequences for the genes. On the other hand, for D1, *uspA1* was ∼2.8 kb, which is larger than the parental 035E *uspA1* (∼2.5 kb), whilst *uspA2* remained unaltered in size at ∼1.6 kb. For D2 *uspA1* was the same size as 035E parental *uspA1* at ∼2.5 kb. However, *uspA2* was ∼1.8 kb, which is larger than the parental 035E *uspA2* (∼1.6 kb). The data suggested that two different insertions had occurred in 035E, one of ∼300 bp in *uspA1* for D1 and another of ∼200 bp in *uspA2* for D2.

**Figure 3:**
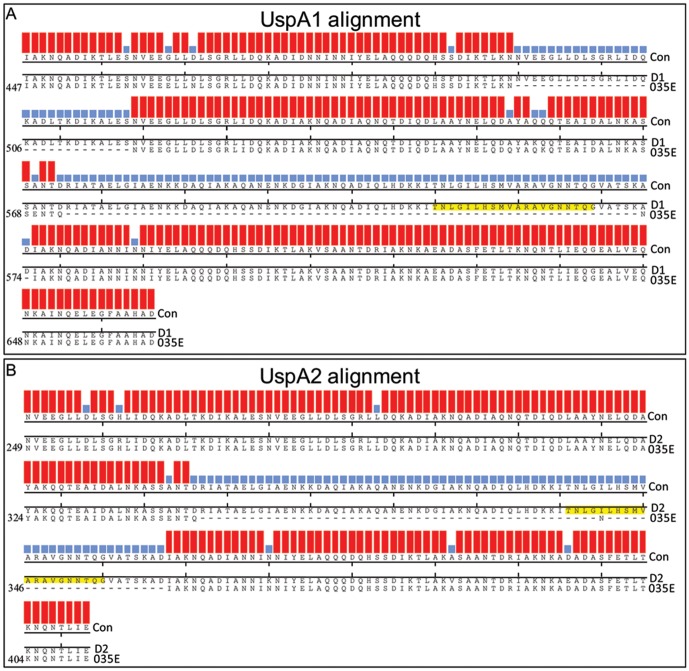
Alignment of UspA variant proteins obtained in strain 035E on transformation with the MX2 *uspA1* gene. Sequences were aligned by pairwise alignment using the ClustalW method within MegAlign DNASTAR software. The overall consensus (Con) strength is shown by coloured histograms above each aligned amino acid; increasing height and colour (light blue–red) indicates increased consensus strength. Sections of alignments are indicated for 035E UspA1 amino acids 447–664 against D1 UspA1 (A). Alignment of 035E UspA2 amino acids 249–412 against D2 UspA2 (B). Note in both cases the insertion of amino acids equivalent to UspA1 of MX2 (defined in the results section) indicated by continuous runs of light blue histogram in each sequence. For reference the amino acid number corresponding to UspA1 (A) and UspA2 (B) from 035E are indicated on the left hand side of each respective alignment. The minimal CEACAM1 binding sequence of UspA1 from MX2 (TNLGILHSMVARAVGNNTQG) is highlighted in yellow.

In order to determine the positions of the insertions, *uspA1* and *uspA2* from D1 and D2 were sequenced. An alignment of the sequence translations confirmed that for D1 the CEACAM binding domain integrated into *uspA1* of 035E whereas for D2 it had integrated into the *uspA2* gene and both of these events led to the gain of CEACAM1 binding function of the previously non-binding 035E proteins (Fig. 1). Given overall homology between the *uspA1* genes, it is not surprising that such recombination would be possible and from sequencing, it was observed that recombination introduced the amino acid 391–617 coding region of MX2 *uspA1* within *uspA1* of 035E at the expense of some of this gene (Fig. 3A). Overall the recombination appeared to lead to a net gain of 99 amino acids which correlates with the increase in PCR product observed (Fig. 2).

For D2, it was clear that recombination had resulted in the introduction of a region of MX2 *uspA1* encoding amino acids 445–653 into 035E *uspA2* again at the expense of some of the original 035E gene resulting in a net gain of 70 amino acids (Fig. 3B). In the case of both D1 and D2, the entire minimal CEACAM1 binding region identified within UspA1 of MX2 (amino acids 578–597, TNLGILHSMVARAVGNNTQG; [27]) has been inserted in either UspA1, or UspA2 of 035E. Outside this region a point mutation resulted in a N601S substitution suggesting this may have been present in the MX2 *uspA1* PCR product. However, and as expected, since this amino acid (S601) falls outside the known CEACAM binding region both derivatives are still able to bind to CEACAM1 (Fig. 1). This is the first time a functional domain has been shown to move between distinct autotransporter adhesins of *M. catarrhalis* by *in vitro* transformation highlighting the potential for rapid development of heterogeneity amongst clinical isolates whilst maintaining functionality needed for colonisation and/or virulence.

### CEACAM binding variants within the Mx population

**Figure 4:**
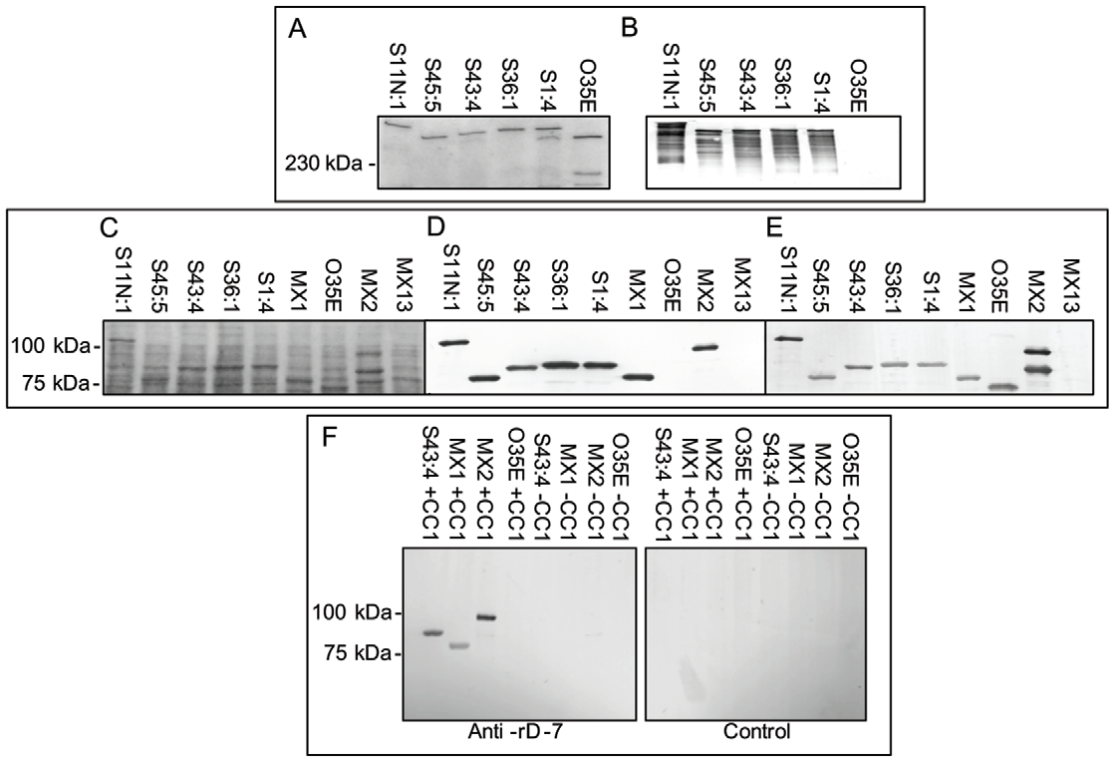
Novel CEACAM-binding proteins of *M. catarrhalis* clinical isolates. Representative Mx strains with novel CEACAM-binding variant proteins were subjected to SDS-PAGE and Western blotting. Gel was stained with Coomassie Blue (A) and corresponding Western blot overlaid with CEACAM1-Fc (B). As observed with 035E D2, the novel CEACAM-binding proteins migrate with a higher apparent molecular weight compared to UspA1 monomers (even after heating of bacterial lysates normally sufficient to dissociate UspA1 into its monomeric form). No CEACAM binding was observed to parental 035E. The laddering effect observed on the CEACAM-binding blot (B) is characteristic of some oligomeric coiled coil adhesins but is not detected by the less sensitive Coomassie Stain (A). (C–E) Several Mx strains were treated with formic acid prior to electrophoresis and the gel after electrophoresis was stained with Coomassie Blue (C) and the corresponding Western blots overlaid with CEACAM1-Fc (D) or anti-rD-7 polyclonal antiserum (E). Following treatment with formic acid, the CEACAM1-binding proteins migrate with higher Mr and react with the antiserum raised against the recombinant polypeptide rD-7 encompassing the UspA1 CEACAM-binding region of Mx strain MX2. No binding of either CEACAM1-Fc or anti-rD-7 was observed to MX13 lacking expression of both UspA1 and UspA2. (F) Western blot showing binding of anti-rD-7 antiserum to the protein co-precipitated using CEACAM1 (+CC1) compared to the control co-precipitation which used protein A- sepharose alone (−CC1). Bands were observed at ∼83 kDa and 90 kDa for MX1 and S43:4 respectively. UspA1 co-precipitated from MX2 migrated at ∼100 kDa and was detected by anti-rD-7 however, no protein detected by anti-rD-7 was co-precipitated from strain 035E. No CEACAM1 co-precipitated proteins were detected by the control antiserum (Control). Whilst regions of interest are presented here, full gel and blot images including Fc and antibody controls are shown in Fig. S2.

Given that the CEACAM1 binding region of UspA1 integrates into UspA2 *in vitro* without any selective pressure, it is possible that such variants occur *in vivo*. To address this possibility, a panel of 100 isolates were screened for CEACAM binding by soluble receptor overlay of Western blots. Of the 100 isolates, 71 bound to CEACAM1. Whilst the majority of isolates possessed CEACAM1-binding proteins which dissociated into monomeric form by heating, suggestive of UspA1 (not shown), 10 of the CEACAM1-binding isolates (14%) possessed higher molecular weight CEACAM binding proteins similar to the CEACAM1-binding protein of 035E D2 (representative isolates are shown in Fig. 4A, B). As observed previously no CEACAM binding to 035E was observed within this region of the blot (Fig. 4A, B).

### Analysis of CEACAM binding variant proteins

In order to confirm if the CEACAM1-binding proteins within these isolates were like UspA2 in biochemical nature, representative novel CEACAM1 binding isolates (S1:4, S11N:1, S36:1, S43:4 and S45:5) were treated with 70% formic prior to electrophoresis and Western blotting. One Mx isolate (MX1) was previously demonstrated to possess a high molecular weight CEACAM1 binding protein [12]. At that time, this protein was considered an anomalous UspA1 protein. In order to assess whether this protein was similar in nature to those under investigation, MX1 was also included within these studies. In contrast to the pattern observed on heating in the absence of formic acid (Fig. 4B), following formic acid treatment, a single CEACAM1 binding protein was observed in each case, the smallest being ∼83 kDa (S45:5 and MX1) and the largest being ∼117 kDa (S11N:1; Fig. 4D). No CEACAM1 binding was observed to the negative control strains 035E and MX13. However, for strain MX2 a ∼100 kDa band was observed binding to CEACAM1 which corresponded to UspA1 (Fig. 4D). The specificity of binding was confirmed using CD33-Fc and SIGLEC1-Fc constructs; which did not bind to any of the CEACAM binding proteins of these strains (Fig. S2). All protein sizes are larger than reported for some previously identified UspA2 proteins with one even larger than expected for some UspA1 proteins (sequencing data to date give a predicted molecular weight range of∼83–96 kDa for mature UspA1 and 60–76 kDa for mature UspA2) [25], [26]. There was an absolute correlation between the binding of CEACAM1-Fc by strains MX1, S1:4, S11N:1, S36:1, S43:4 and S45:5 and that of mouse antiserum raised against rD-7, a recombinant containing the CEACAM1-binding region of MX2 UspA1 (Fig. 4E). In addition, UspA1 of MX2 also bound CEACAM1 and anti-rD-7 (Fig. 4E). Due to the sequence similarity between UspA2 and the non-CEACAM binding region for rD-7, UspA2 of both MX2 and 035E were also detected by anti-rD-7. However, no CEACAM1 or anti-rD-7 binding was observed to MX13 which lacks expression of both UspA1 and UspA2. Further, a protein could be co-precipitated from octyl glucoside extracts of both S43: 4 and MX1 using CEACAM1-Fc (but not from controls lacking CEACAM1) and this ligand was detected using anti-rD-7 antiserum (Fig. 4F) but not control antiserum. UspA1 co-precipitated from MX2 using CEACAM1-Fc was also detected using anti-rD-7; however, no protein detectable by anti rD-7 was co-precipitated from 035E (Fig. 4F). The migration properties of the CEACAM binding variants indicate that UspA2-like proteins possess the CEACAM1 binding motif previously identified only in UspA1 [13]. No strains of Mx appeared to express two bands simultaneously exhibiting CEACAM1-binding, suggesting that strains tested to date either express CEACAM1 binding UspA1 or the novel UspA2-like protein described above but not both.

### Sequence analysis of the variant CEACAM binding proteins

**Figure 5:**
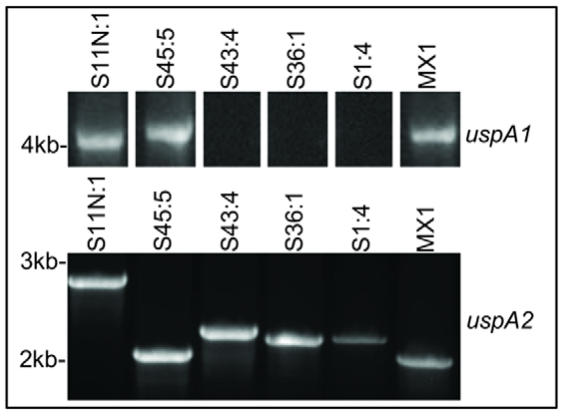
Identification of *uspA* genes from Mx CEACAM-binding variants. PCR of Mx S11N:1, S1:4, S36:1, S43:4, S45:5 and MX1 as indicated for *uspA1* (upper panel) and *uspA2* (lower panel). *uspA1* PCR gave no bands for *uspA1* in strains S1:4, S36:1 and S43:4 whereas larger than expected bands were observed for S11N:1, S45:5 and MX1. PCR products were obtained for *uspA2* for all strains tested. Data are representative of PCR products obtained on several occasions.

Using the same conserved *uspA1* and *uspA2* primers pairs used above, genes were amplified from genomic DNA of strains S1:4, S11N:1, S36:1, S43:4, S45:5 and MX1. For strains S1:4, S36:1 and S43:4 no product was obtained using *uspA1* primer pairs. However, a larger than expected band was obtained for S11N:1 and S45:5 and MX1 at ∼4 kb (Fig. 5). This product is considerably larger than previously identified *uspA1* genes (up to 3.2 kb [26]). Partial sequencing of the *uspA1*-like gene product from MX1 did not identify the known CEACAM binding motif present in UspA1 proteins. However, further studies are underway to obtain the complete sequence of the *uspA1*-like gene from strains MX1 and S11N:1 to ascertain if the known CEACAM binding motif is present. Sequencing will be followed by functional analyses to identify if these *uspA1*-like genes are expressed or not. In the current study, products were obtained for all isolates using the *uspA2* primer pairs (Fig. 5). The relative sizes of the genes correlated well with the relative size of CEACAM1 binding proteins observed in Western blot for each isolate (Fig. 4D). Again, the largest gene product obtained was for S11N:1 (∼2.8 kb) and the smallest was for S45:5 (∼2.1 kb). Given that the novel CEACAM1-binding proteins migrate in a similar fashion to UspA2, and genes of corresponding sizes can be amplified by *uspA2* primer pairs, these novel CEACAM1-binding variant proteins are hereafter referred to as UspA2V (*uspA2V* gene).

**Figure 6:**
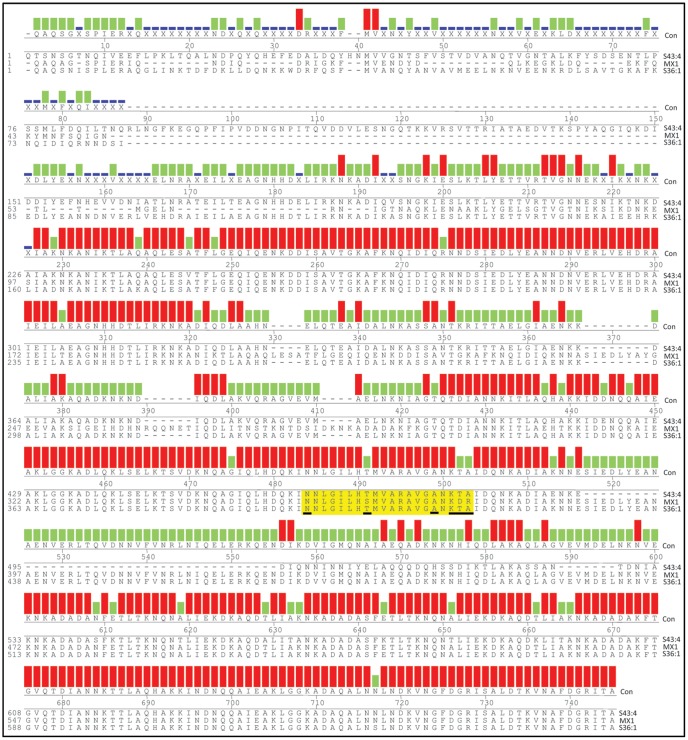
Alignment of UspA2V proteins of representative isolates of Mx. Sequences were aligned by pairwise alignment using the ClustalW alignment method within MegAlign DNASTAR software. Strains are labelled at the right hand side of each sequence line. The top line below the histogram boxes indicates the consensus sequence (Con). The numbering above the alignment indicates positions within the consensus sequence and the numbers to the left hand side refer to the individual sequences. The overall consensus strength is shown by coloured histograms above each aligned amino acid; increasing height and colour indicates increased consensus strength (dark blue<green<red). The CEACAM-binding regions of UspA2V are highlighted in yellow and, within these regions, amino acids differing from the previous sequences identified in UspA1 are underlined.

Five of the *uspA2V* genes (S1:4, S36:1, S43:4, S45:5 and MX1) were sequenced in order to verify the presence of the CEACAM1-binding sequence further. An incomplete sequence for UspA2V from S11N:1 was obtained and therefore this strain was not studied further. UspA2V from MX1 and S45:5 were 99.7% identical at the amino acid level. S1:4 and S36:1 were also 99.7% identical, whilst S43:4 shared the least identity with the other UspA2V proteins (67.0%–76.3%). Thus three distinct sequence types were observed in the five sequences obtained, all of which contained the known CEACAM-binding sequence (Fig. 6). Of the 20 residue region identified spanning amino acids 578–597 of UspA1 from strain MX2 (ATCC25238), the majority were conserved within UspA2V; the exceptions being T>N, N>A and T>K in all sequences (equivalent to T578N, N593A and T595K in UspA1 of MX2). In addition, an S>T (S585T equivalent) was present in strains S1:4, S36:1 and S43:4. Further, Q>T and G>A are present in S1:4, S36:1 and S43:4. Whilst Q>D and G>R replacements are present in MX1 and S45:5 (equivalent to Q596T/D and Q597G/A of MX2, amino acids differing from the CEACAM1 binding domain in UspA1 are underlined in Fig. 6). Whilst not all *uspA2V* genes were sequenced, the presence of the CEACAM1-binding motif in other strains within the UspA2V was shown using the anti-rD-7 antibody overlay of Western blots and by PCR (using the *uspA2* forward primer and a reverse primer located within the CEACAM1-binding coding region of the gene; Fig. S3). Anti-rD-7 bound to a protein of size identical to that bound by CEACAM1 and appropriate PCR products could be obtained for these strains. However, no PCR product was obtained using strains lacking *uspA2V* such as MX2, S39:3 and S13M:3. Thus all UspA2V proteins sequenced possessed the CEACAM1 binding region previously identified and in addition, in each case the key residues identified by previous mutagenesis analyses (i.e. L583, M586, A588 and A590) were absolutely conserved.

### Modular nature of UspA2V

**Figure 7:**
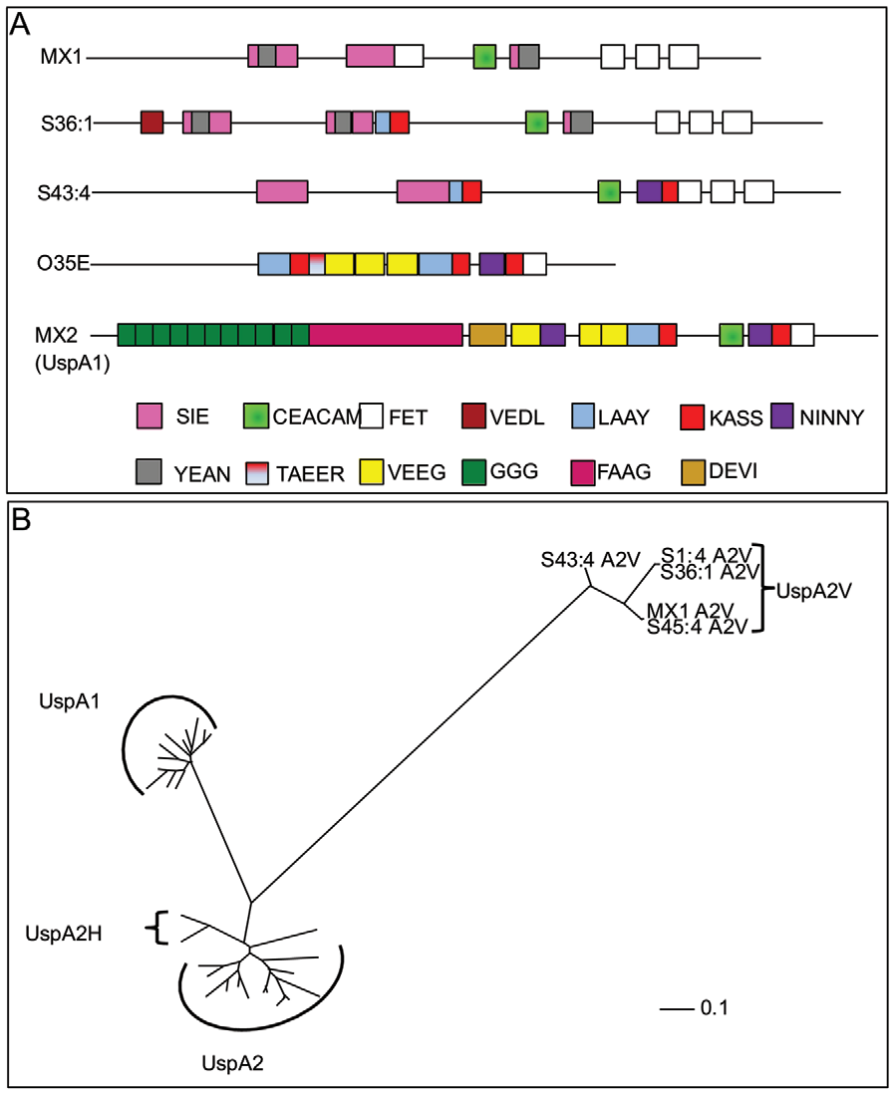
Modular arrangement and phylogenetic relationship of UspA2V proteins. The modular arrangements of UspA2V sequence type obtained are shown for MX1, S36:1 and S43:4 (A). Modules are largely based on those identified in previous work on UspA proteins [25], [26]. Where possible, colour coding has been maintained with previous publications, with the exception that the minimal CEACAM binding domain identified is now included as a distinct refined module [27]. Domains are scaled to indicate relative position and size within each protein sequence shown in Figure 6. Note that all protein sequences identified contain the CEACAM-binding domain as indicated. In addition, schematics for UspA2 of strain 035E and UspA1 of strain MX2 have been included for comparative purposes. B) Phylogenetic relationship of UspA2V proteins. A Phylip Tree file was generated by sequence alignments of UspA2V sequences against all complete UspA proteins in the NCBI protein database using MegAlign software. An unrooted phylogenetic tree was generated by viewing the Phylip Tree file in TreeView software. The UspA2V proteins appear to be distinct from UspA1 and UspA2/H proteins previously sequenced. Clusters of UspA1 and UspA2/H are labelled as indicated rather than individual sequences for clarity of the figure. Scale bar represents nucleotide substitutions per site.

Previous studies of the UspA proteins of Mx have identified numerous repeat motifs within the UspA1, UspA2 and UspA2H [25], [26], [30]. Although the UspA2V proteins were shown above to possess the CEACAM binding motif, the genes could be amplified using conserved *uspA2* primers. We therefore compared their sequences to identify which motifs the A2V proteins possessed (Fig. 7A). All binding functions of the UspA proteins have been attributed to the extracellular domains of the proteins thus the reverse primer used in these studies binds upstream of the region encoding the β-barrel and so this region was not included in this analysis. The N-terminal region of the UspA2V has been trimmed to the first amino acid of the mature protein identified using SignalP. The three distinct A2V proteins share a similar C-terminal region distinct from other UspA proteins in that they contain three FET/FET like regions (Fig. 7A). Upstream of the FET repeat region lie SIE/YEAN regions preceded by the CEACAM binding domain in MX1, S45:5, S36:1 and S1:4. S43:4 is an exception to this in that it possesses an NINNY and KASS motif downstream of the CEACAM binding region. The NINNY motif is present at least once and most commonly twice in all full length UspA proteins described to date [26]. All UspA2V proteins sequenced contain multiple SIE/YEAN motifs in the N-terminal half of the molecule whilst there is a variation in the presence of KASS, LAAY, FET and VEDL motifs between the three different UspA2V protein types sequenced (Fig. 7B). The UspA2V proteins contain domains previously only identified in UspA2 such as SIE, and besides the CEACAM binding region, some contain domains previously only identified in UspA1 (VEDL observed in S36:1 and S1:4). Overall the N-terminal region of UspA2V appears more UspA2-like than UspA1-like, which is also the reason for the successful amplification of the gene with the conserved *uspa2* primer pairs used. It should be noted that UspA2V, whilst possessing known UspA modules, appear to be distinct in modular arrangement compared to previously identified arrangements of UspA proteins in general. An alignment of UspA2V with complete UspA protein sequences deposited in the NCBI protein database revealed that the UspA2V proteins lie in a distinct cluster, further removed from UspA1, UspA2 and UpsA2H than they are from each other. Thus the UspA2V appear to be a distinct and novel group of UspA proteins.

### Cellular interactions of Mx strains expressing novel CEACAM binding UspA proteins

The above studies identified the potential of UspA2V to bind to CEACAMs. However, an important function of UspA2 proteins previously identified is that of vitronectin binding. The latter serves to promote resistance to complement-mediated killing through interference with MAC insertion into the bacterial membranes [24]. It may also be possible that through vitronectin binding, Mx may adhere to host cells by interaction with appropriate integrins. Such a phenomenon has previously been reported for fibronectin interaction with UspA1/A2 [17] but not for vitronectin-mediated binding to integrins. In order to assess if the UspA2V proteins can bind to human epithelial cells via both CEACAMs and integrins, a series of experiments were carried out and are described below.

*Cellular CEACAM binding*: First we used 035E D2 and UspA2V-expressing Mx strains and studied bacterial adhesion to A549 cells by immunofluorescence microscopy. In this case A549 cells were pre-treated with IFN-γ for the following reasons. Inflammatory cytokines, especially IFN-γ, levels increase in patients with COPD [31], [32] and hence target cells treated with IFN-γ mimic the inflammatory state in which COPD lung epithelial cells exist, to some extent. In addition, following such challenge with IFN-γ the expression of CEACAM1, CEA and CEACAM6 has also been shown to increase on A549 and colonic cells [33], [34] and unpublished observations. Mx were added to A549 cells either alone, or preincubated with the CEACAM-binding polyclonal antibody A0115, which by binding to the N-domain of CEACAM1 (and also CEA and CEACAM6) should block CEACAM-mediated binding of Mx to A549 cells. Note that all incubations were performed in the absence of serum to prevent any serum protein (i.e. vitronectin and fibronectin) -mediated integrin binding to the target cells to first define CEACAM1-dependent interactions. Due to the high levels of agglutination afforded by the Hag protein, to facilitate adhesion studies, Hag- mutants of 035E, and D2 were used for the adhesion assays (no Hag mutants could be obtained for clinical isolates in these studies).

**Figure 8:**
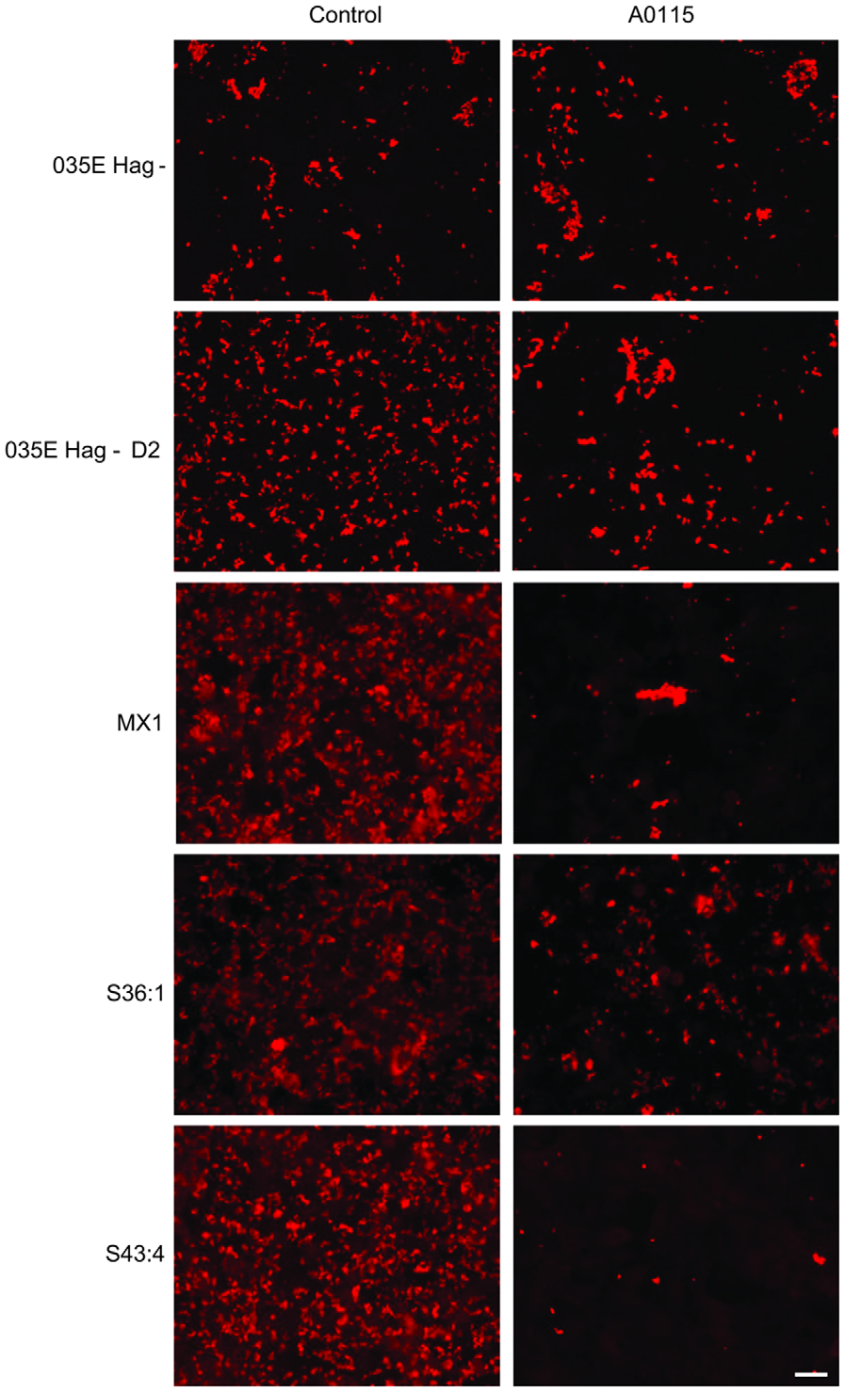
Adherence of the Mx strains expressing CEACAM-binding variant UspA2 proteins to CEACAM1 expressed on A549 human lung epithelial cells. A549 cells were pretreated with IFN-γ for 24 h prior to infection previously shown to upregulate CEACAM1 expression [34]. Cells were infected with 035E Hag-, Hag- derivative of 035E D2 and clinical isolates MX1, S36:1 and S43:4 at an MOI of 100 for 1 hr at RT in medium 199 without serum. Infections were performed without (column 1), or with A0115 (anti-CEACAM binding polyclonal antibody; column 2). Following infection, monolayers were fixed, blocked, and incubated with antisera as described in experimental procedures and rhodamine conjugated secondary antibodies. As can be seen in the right columns, a dramatic reduction in Mx binding in the presence of A0115 occurred in all cases except the Hag- mutant of the 035E parental strain which lacks CEACAM-binding properties. Antibodies not directed against the N-domain of CEACAMs fail to inhibit the interaction of Mx with A549 cells (not shown here; [12]). Data are representative of duplicate infections performed on at least two separate occasions. Scale bar is 20 µm.

Several observations were made from these experiments. Firstly, Mx 035E Hag- D2, possessing CEACAM1 binding motif in UspA2, showed higher levels of binding to A549 cells than observed for the parental UspA2-containing 035E Hag- (Fig. 8). Thus the presence of the CEACAM binding domain in UspA2 conferred improved binding to CEACAM-expressing A549 cells. Secondly, the presence of A0115 did not inhibit 035E Hag- binding to A549 cells, indicating residual CEACAM-independent interaction of 035E Hag- (Fig. 8). Thirdly, for 035E Hag- D2, binding to A549 cells was reduced in the presence of A0115, and the resultant level was similar to that observed for 035E Hag- parental strain. As we have observed previously [12], A549 binding by MX1 (now known to express an UspA2V protein) was reduced in the presence of A0115. In addition, A0115 reduced the binding of UspA2V-expressing S36:1 and S43:4 to A549 cells. Thus the presence of the CEACAM-binding domain, UspA2 (035E D2) or the novel UspA2V (MX1, S36:1 and S43:4) enables Mx to interact with CEACAMs expressed on epithelial cells. Similar results were observed using A549 cells not-stimulated with IFN-γ, although the bacterial binding levels were lower (Data shown in Figure S4, left two columns).

### Vitronectin-mediated cellular integrin binding

**Figure 9:**
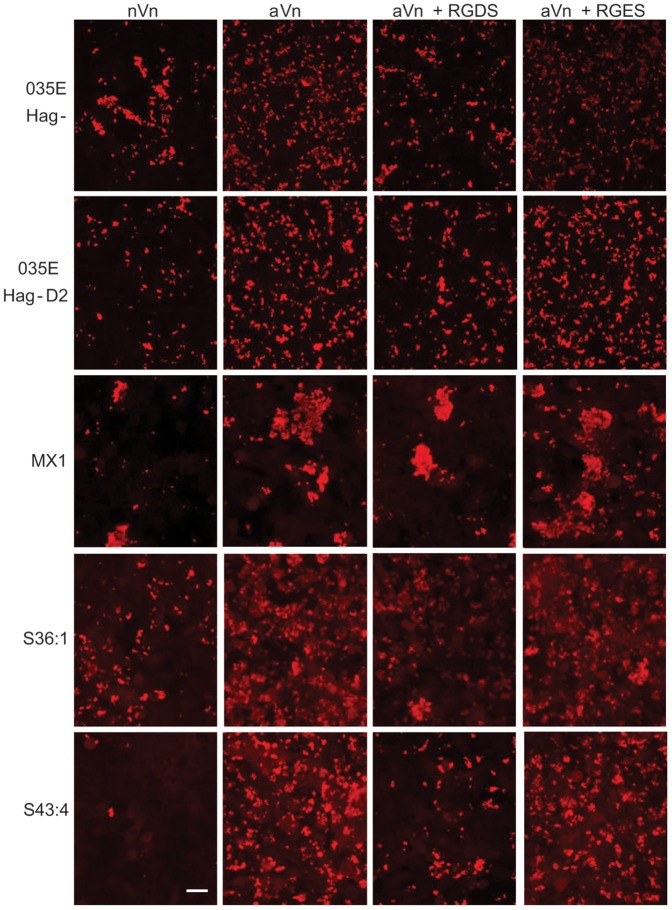
Evaluation of vitronectin-mediated adherence of Mx strains expressing CEACAM-binding UspA2 variant proteins to A549 human lung epithelial cells. A549 cells were pretreated with IFN-γ for 24 h prior to infection to mimic epithelial inflammation. Cells were infected with 035E Hag-, Hag- derivative of 035E D2 and clinical isolates MX1, S36:1 and S43:4 at an MOI of 100 for 1 hr at RT. Infections were performed in medium 199 in the absence of serum but in the presence of A0115 to inhibit CEACAM-mediated interactions (see right column Figure 8 for binding in the absence of A0115). In addition, media contained from the left, native vitronectin (nVn; column 1), activated vitronectin (aVn; column 2), aVn and RGDS (column 3) and aVn and RGES (column 4). Following infection, fixed monolayers were treated as described in the legend to Figure 8. Higher levels of Mx binding in the presence of aVn compared to nVn could be seen in all cases. In addition, a reduction of aVn-mediated Mx binding to A549 cells was observed in the presence of RGDS but not the control tetrapeptide RGES. Data are representative of duplicate infections performed on at least two separate occasions. Scale bar is 20 µm.

Vitronectin has been shown to exist as folded (native; nVn) and open (activated; aVn) conformation within serum [35] and previous studies have shown specific interactions of *Neisseria meningitidis* proteins Opc and Msf (an autotransporter) with aVn [36], [37]. It has been suggested in a recent review that Mx may bind to aVn to a similar extent as nVn [38]. However, their relative roles in UspA2 binding and supporting cellular adhesion are not entirely clear. We also wished to examine if Mx expressing novel UspA proteins with CEACAM binding ability are able to utilise nVn or aVn to adhere to A549 cells. As above, Mx adhesion to IFN-γ-stimulated A549 cells was assessed but the infection medium 199 contained either nVn or aVn. To prevent CEACAM-mediated binding, the antibody A0115 was incorporated in the medium (Fig. 9). The Mx strain 035E Hag- with parental UspA2 demonstrated increased binding to A549 in the presence of aVn but not nVn (Fig. 9). Similar binding levels were observed for D2 in the presence of aVn indicating that acquisition of the CEACAM-binding motif in UspA2 of D2 did not interfere with vitronectin interaction (Fig. 9). The clinical strains expressing UspA2V (MX1, S36:1 and S43:4) in similar fashion, did not show an obvious increase in binding in the presence of nVn. However, increased binding was apparent in the presence of aVn (Fig. 9). The strong agglutination properties of the clinical isolates made quantification of binding difficult, however, the apparent increase in binding supported by aVn was quantified for strain S43:4 by viable colony counting (Fig. S5). For all strains used, inhibition of the aVn-mediated binding was observed in the presence of the integrin-binding tetrapeptide RGDS but not the control peptide RGES suggesting that integrins are involved in mediating Mx interaction with A549 cells via aVn (Fig. 9). Note no inhibitory effect of RGDS alone or in combination with A0115 was observed in the absence of vitronectin (i.e. medium 199 with or without A0115, not shown). In addition, similar results were observed using A549 cells not stimulated with IFN-γ (shown in Fig. S4). Overall the data suggest that Mx strains with novel CEACAM-binding variant UspA2 proteins are able to sustain binding to vitronectin and that activated vitronectin may be favoured and convey a binding advantage over native vitronectin. This is the first time to our knowledge vitronectin has been shown to mediate cellular adherence of Mx. In addition, the binding via vitronectin appears to involve integrins on the surface of epithelial cells as the interaction can be inhibited in the presence of RGDS. Whilst the apparent reduction of binding in the presence of RGDS implicates integrins in mediating Mx adhesion via vitronectin, further studies using specific anti-integrin antibodies are required to elucidate the precise integrins involved in this process. Although the antibody A0115 was used in this instance to fully observe the role of integrins in adhesion, increased aVn-mediated binding of Mx to A549 cells was also observed in the absence of A0115 (Fig. S6). The greater role of aVn versus nVn in supporting adhesion was also verified by assessing if the variant UspA2 expressing Mx bound to aVn to higher levels than nVn (direct binding ELISA shown in Fig. S7). The presence of both CEACAM and vitronectin binding motifs on the same molecule does not appear to impede the binding of Mx to either host protein. Thus, such novel proteins appear to convey an adhesive advantage to the organism and in addition may contribute to serum resistance of these Mx strains.

## Discussion

In this study we have demonstrated that in the absence of selective pressures, the CEACAM-binding region of MX2 *uspA1* can recombine into the *uspA1* gene of strain 035E following *in vitro* transformation conveying the ability to the strain to bind to cellular CEACAM1. Interestingly, the CEACAM binding domain was also shown to be incorporated into UspA2 of 035E. This is the first time this region has been shown to be incorporated into UspA2 and to be present in a variant family UspA2V; the resultant CEACAM1-binding function could be demonstrated in such UspA2 proteins.

These findings suggest that regions of *uspA* genes could be transferred between different *uspA* family genes within the same Mx strain, or following lysis of bacteria *in vivo* which may occur through natural turnover of bacterial cells, the use of antibiotics or through attack by the hosts immune defences. Such domain switching would allow variation amongst the protein family which could serve to aid the avoidance of acquired immunity of the host against other regions of the UspA protein whilst at the same time conserving adhesive and serum resistance functions.

A screen of 100 isolates of Mx identified 14% of CEACAM1 binding isolates tested to possess a CEACAM1 binding protein with UspA2-like properties. Sequencing and phylogenetic analyses also revealed that such proteins not only contained a conserved CEACAM binding motif, but also belonged to a distinct group of UspA proteins which we have termed UspA2V. The discovery of this novel group of proteins raises a number of questions. Firstly, do these proteins represent a truly distinct subgroup of UspA proteins? Based on our detailed analysis of five isolates, this would seem to be the case. However, only a much broader sequencing screen of UspA proteins would reveal the true population structure of the UspA protein without bias that is naturally introduced while using selective screens. Secondly, what are the implications for vaccine design? Phase variation of UspA proteins has been described previously [23], [39]. Such on/off switching could affect the efficacy of any vaccine based on these proteins. Studies have identified the *uspA1* gene/UspA1 protein expression to be present in the majority of strains belonging to phylogenetic group 1 but generally absent in strains identified belonging to group 2 or 3 [22], [23]. The phylogenetic differences identified in these studies also positively correlated with the ability of Mx to adhere to cells (commensurate with high levels of UspA1 expression) and to resist complement-mediated killing which may be linked to UspA2 (group 1) compared to strains that lack adherence (low level of UspA1 expression) and serum sensitivity of group 2 and 3 strains [22]. In this study, vitronectin binding was not examined and there was no correlation of the presence or absence of the *uspA2* gene between serum resistant and serum-sensitive groups; although it should be noted that protein expression was not examined. MLST-based analyses found higher rates of recombination and mutation within house keeping genes of the serum resistant group compared to the serum-sensitive group suggesting that recombinant events in general may well be higher in Mx strains belonging to group 1 [40]. This study also acknowledged that the dichotomy was not perfect with 86% of serum-sensitive isolates being assigned to the serum-sensitive group [40]. Of the isolates shown in our current study to possess UspA2V, 16S rRNA sequencing revealed them to belong to phylogenetic groups 2/3 (data not shown). However, it is possible that these isolates could represent a minority proportion within these phylogenetic grouping and a further screen would be required to prove otherwise. The findings of this study demonstrate that despite belonging to phylogenetic groups 2 and 3, the strains expressing UspA2V are able to interact not only with cell-expressed CEACAMs but also mediate cell attachment via interaction with vitronectin. Although without a knockout of UspA2V, we cannot state categorically that CEACAM and vitronectin binding are solely due to UspA2V expression by the strains examined, we have other evidence which suggests this may be the case. A recombinant protein of UspA2V derived from strain S43:4 showed binding to both CEACAM1 and vitronectin in a dose dependent manner (Fig. S8). Thus it appears that UspA2V is able to mediate both CEACAM1 and vitronectin binding. As the group 2/3 strains are thought to be older than the group 1 strains in evolutionary terms and less virulent [40], our findings suggest that strains expressing UspA2V could be a link in the evolution of the more virulent group 1 Mx. Alternatively, they could represent a separate branch that may have arisen from recombinant events between group 1 and group 2/3 strains. It is therefore interesting to note that despite belonging to group 2/3 a number of strains, from which *uspA2V* was sequenced in this study, were isolated from patients rather than from asymptomatic carriers, indicating that they can be virulent. In the current study we were unable to knock out either *hag* or *uspA2V* using constructs which we have successfully used for 035E in this study for Hag and other studies for UspA2 (Hill and Virji unpublished observations). Knockouts were attempted using both natural transformation or electroporation techniques. Whilst the precise reason for the lack of transformants remains to be determined, this may be due to sequence differences between existing knockout constructs and the sequences within and flanking the genes in question. Whole genome sequencing of strains expressing UspA2V may not only address this matter, but also yield information regarding the relationship of these strains with other MX isolates. For example, previous studies have identified that strains of Mx express either UspA2 or UspA2H but not both proteins [30]. Whilst only a single gene product was obtained using *uspA2* primers from clinical isolates used in the current study, whole genome sequencing will identify the presence of other *uspA*-related genes or pseudogenes within these strains.

To our knowledge, this is the first report of Mx interaction with cellular integrins via vitronectin and of a clear preference for binding to activated form of vitronectin. Previously, binding to vitronectin has been shown to enhance serum resistance [24], [38]. Thus the UspA2V proteins not only convey two independent means of Mx adhesion to epithelial cells (to CEACAMs and to integrins via aVn), but via vitronectin binding they also have the potential to control a critical stage of complement function by inhibiting the complement membrane attack complex.

Interestingly, previous studies have identified distinct cluster of *uspA2* genes belonging to group 2/3 [23], however, further functional investigations on such gene products have not been reported. The discovery of this novel group of UspA proteins indicates the need for further studies in order to understand the population dynamics relating to UspA proteins. It is interesting to speculate that variation among the UspA proteins is driven by the immune challenges faced by Mx during colonisation and pathogenesis. Studies on *H. influenzae* have identified antigenic variation in the outer membrane protein P5 (also known to bind to CEACAMs) in prolonged carriage during chronic bronchitis. [14], [41]. Such variation within P5 clearly shows a selective advantage within these bacteria. Individual residues within autotransporters are also subject to immune pressure. For example, individual amino acids have been identified within the autotransporter adhesin pertactin of *Bordetella* species which are subject to positive selective pressure, leading to changes which may influence antibody recognition and immune evasion [42]. Although in both cases the studies identify antigenic drift within strains rather than switching of whole functional domains between strains/related proteins. Similar to *H. influenzae*, longitudinal carriage of Mx has been demonstrated [43], [44] and variation of UspA1 and UspA2/H proteins in different carriage isolates from children have been reported [45]. However, no studies appear to have examined *in vivo* variation of UspA proteins, either by antigenic drift or domain switching, during continuous longitudinal carriage or in disease conditions such as COPD exacerbation, the current study highlights the potential for rapid homologous recombination to occur during multiple strain carriage.

Considering *in vivo* situations, it is pertinent to examine environmental factors that alter bacterial phenotype. For example, UspA1 expression has been shown to increase following exposure of Mx to temperatures mimicking those of the nasopharynx in colder climates/seasons irrespective of phylogenetic group [46]. In addition, to UspA1, RecA, the enzyme responsible for homologous recombination within the Mx genome, is also upregulated at such temperatures [47]. Thus bacterial ability to recombine and produce adhesive specificities for targeting novel host tissues may be enhanced. *In vivo*, the host target tissues may also present a greater density of cell surface receptors, such situations arise following inflammation. Increased production of inflammatory cytokines such as IFN-γ (often observed following viral infection [48]) can upregulate CEACAM expression on epithelial cells and lead to increased bacterial adhesion and invasion [34], [48]. Whilst the latter study examined adhesion and invasion of *Neisseria meningitidis*, it is possible that a similar scenario exists for other CEACAM-binding pathogens such as Mx and Hi, both of which also show increased carriage rates during winter months and increased propensity to cause disease [49]. Increased carriage, however, may also enhance the probability of increased transformation *in vivo*, and exchange of virulence properties within the carriage population. Thus both environmental factors (such as temperature) and host factors (such as prior inflammation) are likely to drive antigenic variation of UspA proteins *in vivo*, leading to new emergent phenotypes with heightened pathogenic potential. Whilst our studies have focussed on autotransporter proteins from Mx, it is possible that similar recombination events leading to the transfer of functional motifs between related autotransporter family members could occur in other species. For example *Bartonella quintana* expresses up to 4 members of the variably expressed outer membrane protein (Vomp) family of autotransporters. These autotransporter proteins are associated with adhesion to host cells and extracellular matrix proteins. However, not all Vomps function as ECM adhesins suggesting the acquisition or loss of functional domains by proteins within this family [50].

Autotransporters have proven successful as vaccine components, with pertactin showing the highest correlation with protection against *Bordetella pertussis* [51]. UspA proteins have long been considered potential vaccine candidates and a common motif has been described which leads to protection against Mx infection [11]. Thus bacterial adhesins, including autotransporters, present the bacteria with a “double edged sword” whereby maintaining adhesion needs to be balanced against immune evasion during evolution [52]. The current study identifies a functional motif previously thought only to be present in UspA1 proteins, to be present also in a novel variant of UspA2 (UspA2V). Targeting conserved motifs of known function present in multiple proteins such as the CEACAM binding domain or domains responsible for interaction with matrix proteins such as vitronectin and fibronectin, may prove a useful strategy for vaccination; particularly as certain functional domains may be necessary/contribute to pathogenesis and colonisation and without which organisms would be less likely to cause disease. We have previously shown the CEACAM binding motif of UspA proteins to be immunogenic and have adhesive blocking functions [13]. Overall, these studies identify the need for further investigation into the vaccine potential of UspA protein regions such as the CEACAM binding domain. In addition, broader phylogenetic analyses of the UspA proteins are required to further our understanding of the antigenic variation within this protein family in order to inform future therapeutic strategies.

## Experimental Procedures

### Bacterial isolates and culture

Mx isolates were grown on brain heart infusion (BHI) agar (Oxoid Ltd, UK) supplemented with 10% heated horse blood (TCS Biosciences Ltd, UK). All bacteria were cultured at 37°C for 16–20 h in a 5% CO_2_ incubator unless otherwise indicated. To screen clinical and carriage isolates for CEACAM binding strains, obtained from either healthy volunteers or patients presenting with cases of otitis media and COPD (approximately two thirds of the isolates used were from patients). Isolates were obtained from several contributors including Dr Anges Wold and Miss Susann Skovjberg, Sahlgrenska University Hospital, Goteborg, Sweden. Professor Mark Achtman University of Cork, Ireland [40] (see http://mlst.ucc.ie/ for MLST data) and Professor Tone Tonjum Oslo University Hospital, Oslo, Norway. Other strains including MX1, MX2 and 035E have been described in previous studies [12], [27]. MX13 is a variant derived from ATCC25240 lacking expression of both UspA1 and UspA2. Further information on the strains used for more detailed analyses in this study is provided in Table 1.

**Table 1:** Otitis media or COPD-associated isolates of Mx strains used in this study.

Strain Name	Site of isolation
035E	Middle ear fluid
MX2	ATCC25238 (type strain)
MX1	Sputum
S11N:1	Nasopharynx
S1:4	Nasopharynx
S36:1	Nasopharynx
S43:4	Nasopharynx
S45:5	Nasopharynx

### Antibodies

Polyclonal antiserum against Mx was raised in rabbits using standard protocols and whole cell lysates of multiple strains as antigens. Anti-rD-7 antiserum was generated in mice by immunisation with unconjugated polypeptide [(rD-7) 25–50 µg per immunisation)]. All antisera were generated as a service within the University of Bristol under a home office granted antibody service project license. A0115 raised against human CEA (also binds to CEACAM1 and CEACAM6) was obtained from Dako (code no. A0115). The antibody has been shown to inhibit Mx interactions with CEACAM1 previously [12].

### Soluble receptor constructs and cell lines

Soluble CEACAM1-Fc has been described previously [53]. A549 human lung carcinoma cells (Flow laboratories) were cultured in F12 Ham medium containing 10% foetal calf serum (FCS; Lonza, UK).

### Bacterial transformation with *uspA1*

Genomic DNA was purified from MX2 using a DNeasy Tissue Kit (Qiagen) and uspa1 amplified by polymerase chain reaction (PCR) using uspa1F-uspa1R primer pair. The resulting product was cloned in pGEMTeasy (Clonetech). Purified pGEMTeasy-*uspA1* was linearised with *Pst*I and used to transform naturally competent 035E according to previously described methods [54] with the exception that 1 µg of DNA was used in the transformation.

### Colony blotting

Colonies of 035E following transformation were plated onto HBHI agar at ∼1000–2000 colony forming units (cfu) per plate and grown overnight at 37°C for 16–20 h in a 5% CO_2_ incubator. Colonies were lifted onto nitrocellulose and nonspecific binding sites blocked with 5% milk in phosphate buffered saline containing 0.05% Tween-20 (PBS-T). Colony blots were overlaid with CEACAM1-Fc (1 µg. ml^−1^) and CEACAM binding detected using anti-human-Fc alkaline phosphatase conjugated antibodies.

### 035E Hag knockout mutants

In order to reduce bacterial autoagglutination during cell adhesion assays, Hag was knocked out of 035E and variants D1 and D2 by the following method. The *hag* gene was amplified by PCR using HagF-HagR primer pairs (Table 2) and cloned into pGEMTeasy. PGEMTeasy-*hag* was digested with *Mfe*I and ligated with the Kanamycin resistance cassette excised by *EcoR*I digestion of pUC4K. Insertion of kanamycin cassette at this position will disrupt Hag beyond amino acid 69 of the gene product. The Hag knockout construct was used to transform naturally competent Mx as described above. Hag knock out mutants were selected by growth on HBHI containing 20 µg. ml^−1^ kanamycin. Hag knockout was confirmed by SDS-PAGE and spectrophotometric agglutination assay (data not shown). Although variable aggregation was observed for the other clinical isolates used within this study no Hag knockout mutants could be generated using the same construct by either natural transformation or electroporation.

**Table 2:** Primer names and sequences used in this study.

Primer Name	Sequence (3′-5′)
uspa1F	5′-AATGCCGCAGGTCACTTG-3′
uspa1R	5-′TTTCCAGCGGTAACTGCC-3′
uspa2F	5-′GAAAACCATGAAACTTCTCC-3′
uspa2R	5-′ATAAGGCTGGAATAGACC-3′
D-7R	5-′GTGTGTTATTTCCTACCTGTCTTGCAACCATGCTGTGAAGG-3′
HagF	5-′AATCACATCTATAAAGTC-3′
HagR	5-′AAAGTGAAAACCTGCACC-3′

### Immunoprecipitation of the ligand with CEACAM1-Fc

To capture the bacterial CEACAM-binding ligand, 100 µl of protein A (50% slurry) coupled to sepharose CL-4B (Sigma) was incubated with 20 µg of CEACAM1-Fc overnight at 4°C and subsequently washed three times with PBSB (Dulbecco's complete PBS) to remove any unbound receptor. Simultaneously, overnight cultures of bacteria were suspended in 100 mM octyl-β-d-glucopyranoside (OG; final concentration) in PBSB containing protease inhibitor cocktail (PIC). Samples were mixed end-over-end overnight at 4°C. Insoluble bacterial material was removed by centrifugation at 10,000 *g* for 15 min. Soluble supernatant was divided in two 500 µl aliquots and incubated with either CEACAM1-Fc–Protein A sepharose complex or the control (protein A-sepharose alone) for 3 h at 4°C. After washing 3 times with 50 mM OG and PBSB, samples were dissociated with Formic acid as described below prior to electrophoresis and Western blotting.

### SDS-PAGE and Western blotting

Bacterial lysates were prepared in SDS-PAGE dissociation buffer and heated at 100°C for 10 min. Where indicated, bacterial lysates were pretreated with 70% formic acid (v/v) and incubated overnight at room temperature in the dark. The sample was then freeze dried, resuspended in an appropriate volume of sample buffer and either heated at 100°C or left at RT for 10 min. Whole-cell lysates of Mx (∼3×10^7^ per lane) were applied to 7.5% Tris-Glycine polyacrylamide gels and run under standard conditions. Proteins from the gels were transferred to nitrocellulose membranes, and the membranes were overlaid with CEACAM1-Fc (1 µg. ml^−1^) to assess receptor binding which was detected as described under colony blotting. Alternatively, blots were overlaid with anti-rD-7 antiserum to detect the presence of the CEACAM-binding motif and its binding detected with anti-mouse-alkaline phosphatase conjugated secondary antibodies.

### PCR and sequencing

Genomic DNA was extracted from Mx isolates using a DNeasy Tissue Kit (Qiagen) according to the manufacturer's instructions. Genes were amplified using uspa1 and uspa2 primer pairs (Table 2) purified using a PCR cleanup kit (Qiagen). Genes were sequenced through the DNA sequencing service (http://www.dnaseq.co.uk), using appropriate forward and reverse primers, as well as primer D7R to ensure coverage. Sequence analysis and alignment was performed using Lasergene EditSeq, SeqBuilder and MegAlign software. Phylogenetic relationships were viewed using TreeView following alignment in MegAlign. Sequence data have been submitted to the GenBank database under accession numbers JF706365 (MX1), JF706366 (S1:4), JF706367 (S36:1), JF706368 (S45:5) and JF706369 (S43:4).

### Bacterial adherence to A549 cells

Confluent monolayers of A549 cells were, where indicated, preincubated with IFN-γ (100 U. ml^−1^; Sigma) in Medium 199 (Sigma) for 24 h. Prior to infection, cells were preincubated with A0115 (100 µg. ml^−1^ total Ig purified from rabbit serum) for 30 min at 37°C in medium 199 where indicated. Subsequently, monolayers were incubated with ∼100 bacteria per cell in medium 199 without FCS (Lonza, UK). For studies on the role of vitronectin, the infection medium 199 was supplemented with nVn (10 µg. ml^−1^ Molecular Innovations) or aVn (10 µg. ml^−1^ Sigma). For inhibition of integrin binding, monolayers were preincubated with the peptide RGDS, RGES was used as a negative control (both 0.25 mM) for 30 min at 37°C in medium 199 prior to infection. Non-adherent bacteria were removed by washing 4 times with medium 199 and for immunofluorescence microscopy, monolayers were fixed with 2% paraformaldehyde. The monolayers were subsequently washed and blocked with 3% bovine serum albumin in PBS containing 0.05% Tween 20 for 1 h. Adherent bacteria were detected using either specific antisera raised against Mx or using normal human serum which proved suitable for the detection of most of the isolates. Finally, rhodamine conjugated secondary antibodies were used and monolayers were examined using an Olympus IX70 microscope, with ×400 magnification.

### ELISA and immunodotblot assays

ELISA plates (96 well, Dynex) were coated with whole cell bacterial lysates of Mx by overnight incubation in carbonate buffer pH 9.5 (∼1×10^9^ bacteria. ml^−1^). Either aVn or nVn (both 2 µg. ml^−1^ in PBS) were incubated with immobilised bacterial lysates for 1 h at RT. Vitronectin binding was detected using polyclonal antiserum raised against Vn, followed by AP-conjugated secondary antibody. All antibodies were incubated for 1 h at room temperature in 1% BSA-PBS. ELISA plates were developed using SigmaFast p-Nitrophenyl phosphate substrate and absorbance was measured at 405 nm (A405).

For immunodotblot overlay, Mx lysates were immobilised onto nitrocellulose membrane, blocked with 3% BSA in PBS and overlaid with aVn or nVn (2 µg. ml^−1^). To remove any aVn from nVn preparations, samples were pre-incubated for 1 h with heparin-agarose (heparin depleted nVn). To activate nVn by unfolding, nVn preparations were heated at 56°C for 30 min (heated nVn). Vitronectin binding was detected with rabbit polyclonal antisera and AP-conjugated secondary antibodies. Relative levels of antibody binding were ascertained by densitometry using Scion Image software (Scion Corporation, Maryland USA).

## Supporting Information

Figure S1 **CEACAM1 binding properties of** ***M. catarrhalis*** **strain 035E and its derivatives.**
(PDF)

Figure S2 **Novel CEACAM-binding proteins of** ***M. catarrhalis*** **clinical isolates.**
(PDF)

Figure S3 **Western Blot and PCR Analyses of the** ***M. catarrhalis*** **strains expressing UspA2 variant proteins not analysed further in the manuscript.**
(PDF)

Figure S4 **Adhesion of the** ***M. catarrhalis*** **strains expressing UspA2 variant proteins to A549 human lung epithelial cells via CEACAM or vitronectin.**
(PDF)

Figure S5 **Vitronectin-mediated adherence of Mx strain S43:4 expressing the UspA2 variant protein to A549 human lung epithelial cells.**
(PDF)

Figure S6 **Simultaneous CEACAM and vitronectin-mediated adherence of Mx strains expressing the UspA2 variant proteins to A549 human lung epithelial cells.**
(PDF)

Figure S7 **Binding of vitronectin directly to bacteria expressing variant UspA proteins.**
(PDF)

Figure S8 **Binding of CEACAM1 and vitronectin directly to recombinant UspA2V.**
(PDF)

## References

[1] Murphy TF, Parameswaran GI (2009) *Moraxella catarrhalis*, a human respiratory tract pathogen. Clin Infect Dis 49: 124–131. 19480579 10.1086/599375

[2] Cripps AW, Otczyk DC, Kyd JM (2005) Bacterial otitis media: a vaccine preventable disease? Vaccine 23: 2304–2310. 15755616 10.1016/j.vaccine.2005.01.023

[3] Parameswaran GI, Wrona CT, Murphy TF, Sethi S (2009) *Moraxella catarrhalis* acquisition, airway inflammation and protease-antiprotease balance in chronic obstructive pulmonary disease. BMC Infect Dis 9: 178. 19912665 10.1186/1471-2334-9-178 PMC2780445

[4] Mannino DM, Buist AS (2007) Global burden of COPD: risk factors, prevalence, and future trends. Lancet 370: 765–773. 17765526 10.1016/S0140-6736(07)61380-4

[5] de Vries SP, van Hijum SA, Schueler W, Riesbeck K, Hays JP, et al (2010) Genome analysis of *Moraxella catarrhalis* strain RH4, a human respiratory tract pathogen. J Bacteriol 192: 3574–3583. 20453089 10.1128/JB.00121-10 PMC2897349

[6] de Vries SP, Bootsma HJ, Hays JP, Hermans PW (2009) Molecular aspects of *Moraxella catarrhalis* pathogenesis. Microbiol Mol Biol Rev 73: 389–406. 19721084 10.1128/MMBR.00007-09 PMC2738133

[7] Cotter SE, Surana NK, St Geme JW 3rd (2005) Trimeric autotransporters: a distinct subfamily of autotransporter proteins. Trends Microbiol 13: 199–205. 15866036 10.1016/j.tim.2005.03.004

[8] Aebi C, Maciver I, Latimer JL, Cope LD, Stevens MK, et al (1997) A protective epitope of *Moraxella catarrhalis* is encoded by two different genes. Infect Immun 65: 4367–4377. 9353007 10.1128/iai.65.11.4367-4377.1997 PMC175628

[9] Helminen ME, Maciver I, Latimer JL, Klesney-Tait J, Cope LD, et al (1994) A large, antigenically conserved protein on the surface of *Moraxella catarrhalis* is a target for protective antibodies. J Infect Dis 170: 867–872. 7523537 10.1093/infdis/170.4.867

[10] Aebi C, Lafontaine ER, Cope LD, Latimer JL, Lumbley SL, et al (1998) Phenotypic effect of isogenic uspA1 and uspA2 mutations on *Moraxella catarrhalis* 035E. Infect Immun 66: 3113–3119. 9632574 10.1128/iai.66.7.3113-3119.1998 PMC108321

[11] McMichael JC, Fiske MJ, Fredenburg RA, Chakravarti DN, VanDerMeid KR, et al (1998) Isolation and characterization of two proteins from *Moraxella catarrhalis* that bear a common epitope. Infect Immun 66: 4374–4381. 9712790 10.1128/iai.66.9.4374-4381.1998 PMC108528

[12] Hill DJ, Virji M (2003) A novel cell-binding mechanism of *Moraxella catarrhalis* ubiquitous surface protein UspA: specific targeting of the N-domain of carcinoembryonic antigen-related cell adhesion molecules by UspA1. Mol Microbiol 48: 117–129. 12657049 10.1046/j.1365-2958.2003.03433.x

[13] Hill DJ, Edwards AM, Rowe HA, Virji M (2005) Carcinoembryonic antigen-related cell adhesion molecule (CEACAM)-binding recombinant polypeptide confers protection against infection by respiratory and urogenital pathogens. Mol Microbiol 55: 1515–1527. 15720557 10.1111/j.1365-2958.2005.04487.x

[14] Hill DJ, Toleman MA, Evans DJ, Villullas S, Van Alphen L, et al (2001) The variable P5 proteins of typeable and non-typeable *Haemophilus influenzae* target human CEACAM1. Mol Microbiol 39: 850–862. 11251807 10.1046/j.1365-2958.2001.02233.x

[15] Virji M, Watt SM, Barker S, Makepeace K, Doyonnas R (1996) The N-domain of the human CD66a adhesion molecule is a target for Opa proteins of *Neisseria meningitidis* and *Neisseria gonorrhoeae* . Mol Microbiol 22: 929–939. 8971714 10.1046/j.1365-2958.1996.01548.x

[16] Hammarstrom S (1999) The carcinoembryonic antigen (CEA) family: structures, suggested functions and expression in normal and malignant tissues. Semin Cancer Biol 9: 67–81. 10202129 10.1006/scbi.1998.0119

[17] Tan TT, Nordstrom T, Forsgren A, Riesbeck K (2005) The respiratory pathogen *Moraxella catarrhalis* adheres to epithelial cells by interacting with fibronectin through ubiquitous surface proteins A1 and A2. J Infect Dis 192: 1029–1038. 16107956 10.1086/432759

[18] Tan TT, Forsgren A, Riesbeck K (2006) The respiratory pathogen *Moraxella catarrhalis* binds to laminin via ubiquitous surface proteins A1 and A2. J Infect Dis 194: 493–497. 16845633 10.1086/505581

[19] Manolov T, Forsgren A, Riesbeck K (2008) Purification of alpha1-antichymotrypsin from human plasma with recombinant *M. catarrhalis* ubiquitous surface protein A1. J Immunol Methods 333: 180–185. 18242635 10.1016/j.jim.2007.12.012

[20] Nordstrom T, Blom AM, Tan TT, Forsgren A, Riesbeck K (2005) Ionic binding of C3 to the human pathogen *Moraxella catarrhalis* is a unique mechanism for combating innate immunity. J Immunol 175: 3628–3636. 16148107 10.4049/jimmunol.175.6.3628

[21] Nordstrom T, Blom AM, Forsgren A, Riesbeck K (2004) The emerging pathogen *Moraxella catarrhalis* interacts with complement inhibitor C4b binding protein through ubiquitous surface proteins A1 and A2. J Immunol 173: 4598–4606. 15383594 10.4049/jimmunol.173.7.4598

[22] Bootsma HJ, van der Heide HG, van de Pas S, Schouls LM, Mooi FR (2000) Analysis of *Moraxella catarrhalis* by DNA typing: evidence for a distinct subpopulation associated with virulence traits. J Infect Dis 181: 1376–1387. 10762569 10.1086/315374

[23] Meier PS, Troller R, Heiniger N, Grivea IN, Syrogiannopoulos GA, et al (2005) *Moraxella catarrhalis* strains with reduced expression of the UspA outer membrane proteins belong to a distinct subpopulation. Vaccine 23: 2000–2008. 15734074 10.1016/j.vaccine.2004.09.036

[24] Attia AS, Ram S, Rice PA, Hansen EJ (2006) Binding of vitronectin by the *Moraxella catarrhalis* UspA2 protein interferes with late stages of the complement cascade. Infect Immun 74: 1597–1611. 16495531 10.1128/IAI.74.3.1597-1611.2006 PMC1418666

[25] Cope LD, Lafontaine ER, Slaughter CA, Hasemann CA Jr, Aebi C, et al (1999) Characterization of the *Moraxella catarrhalis* uspA1 and uspA2 genes and their encoded products. J Bacteriol 181: 4026–4034. 10383971 10.1128/jb.181.13.4026-4034.1999 PMC93893

[26] Brooks MJ, Sedillo JL, Wagner N, Laurence CA, Wang W, et al (2008) Modular arrangement of allelic variants explains the divergence in *Moraxella catarrhalis* UspA protein function. Infect Immun 76: 5330–5340. 18678659 10.1128/IAI.00573-08 PMC2573364

[27] Conners R, Hill DJ, Borodina E, Agnew C, Daniell SJ, et al (2008) The Moraxella adhesin UspA1 binds to its human CEACAM1 receptor by a deformable trimeric coiled-coil. EMBO J 27: 1779–1789. 18497748 10.1038/emboj.2008.101 PMC2396876

[28] Brooks MJ, Sedillo JL, Wagner N, Wang W, Attia AS, et al (2008) *Moraxella catarrhalis* binding to host cellular receptors is mediated by sequence-specific determinants not conserved among all UspA1 protein variants. Infect Immun 76: 5322–5329. 18678656 10.1128/IAI.00572-08 PMC2573313

[29] Klingman KL, Murphy TF (1994) Purification and characterization of a high-molecular-weight outer membrane protein of *Moraxella (Branhamella) catarrhalis* . Infect Immun 62: 1150–1155. 8132320 10.1128/iai.62.4.1150-1155.1994 PMC186244

[30] Lafontaine ER, Cope LD, Aebi C, Latimer JL, McCracken GH Jr, et al (2000) The UspA1 protein and a second type of UspA2 protein mediate adherence of *Moraxella catarrhalis* to human epithelial cells in vitro. J Bacteriol 182: 1364–1373. 10671460 10.1128/jb.182.5.1364-1373.2000 PMC94425

[31] Brozyna S, Ahern J, Hodge G, Nairn J, Holmes M, et al (2009) Chemotactic mediators of Th1 T-cell trafficking in smokers and COPD patients. Copd 6: 4–16. 19229703 10.1080/15412550902724164

[32] Di Stefano A, Caramori G, Capelli A, Gnemmi I, Ricciardolo FL, et al (2004) STAT4 activation in smokers and patients with chronic obstructive pulmonary disease. Eur Respir J 24: 78–85. 15293608 10.1183/09031936.04.00080303

[33] Fahlgren A, Baranov V, Frangsmyr L, Zoubir F, Hammarstrom ML, et al (2003) Interferon-gamma tempers the expression of carcinoembryonic antigen family molecules in human colon cells: a possible role in innate mucosal defence. Scand J Immunol 58: 628–641. 14636419 10.1111/j.1365-3083.2003.01342.x

[34] Griffiths NJ, Bradley CJ, Heyderman RS, Virji M (2007) IFN-gamma amplifies NFkappaB-dependent *Neisseria meningitidis* invasion of epithelial cells via specific upregulation of CEA-related cell adhesion molecule 1. Cell Microbiol 9: 2968–2983. 17764466 10.1111/j.1462-5822.2007.01038.x PMC3020365

[35] Izumi M, Yamada KM, Hayashi M (1989) Vitronectin exists in two structurally and functionally distinct forms in human plasma. Biochim Biophys Acta 990: 101–108. 2465025 10.1016/s0304-4165(89)80019-4

[36] Griffiths NJ, Hill DJ, Borodina E, Sessions RB, Devos NI, et al (2011) Meningococcal surface fibril (Msf) binds to activated vitronectin and inhibits the terminal complement pathway to increase serum resistance. Mol Microbiol 82: 1129–1149. 22050461 10.1111/j.1365-2958.2011.07876.x

[37] Sa E, Cunha C, Griffiths NJ, Virji M (2010) *Neisseria meningitidis* Opc invasin binds to the sulphated tyrosines of activated vitronectin to attach to and invade human brain endothelial cells. PLoS Pathog 6: e1000911. 20502634 10.1371/journal.ppat.1000911 PMC2873925

[38] Singh B, Su YC, Riesbeck K (2010) Vitronectin in bacterial pathogenesis: a host protein used in complement escape and cellular invasion. Mol Microbiol 78: 545–560. 20807208 10.1111/j.1365-2958.2010.07373.x

[39] Lafontaine ER, Wagner NJ, Hansen EJ (2001) Expression of the *Moraxella catarrhalis* UspA1 protein undergoes phase variation and is regulated at the transcriptional level. J Bacteriol 183: 1540–1551. 11160084 10.1128/JB.183.5.1540-1551.2001 PMC95038

[40] Wirth T, Morelli G, Kusecek B, van Belkum A, van der Schee C, et al (2007) The rise and spread of a new pathogen: seroresistant *Moraxella catarrhalis* . Genome Res 17: 1647–1656. 17895425 10.1101/gr.6122607 PMC2045147

[41] Duim B, Bowler LD, Eijk PP, Jansen HM, Dankert J, et al (1997) Molecular variation in the major outer membrane protein P5 gene of nonencapsulated *Haemophilus influenzae* during chronic infections. Infect Immun 65: 1351–1356. 9119473 10.1128/iai.65.4.1351-1356.1997 PMC175139

[42] Diavatopoulos DA, Hijnen M, Mooi FR (2006) Adaptive evolution of the *Bordetella* autotransporter pertactin. J Evol Biol 19: 1931–1938. 17040390 10.1111/j.1420-9101.2006.01154.x

[43] Faden H, Harabuchi Y, Hong JJ (1994) Epidemiology of *Moraxella catarrhalis* in children during the first 2 years of life: relationship to otitis media. J Infect Dis 169: 1312–1317. 8195609 10.1093/infdis/169.6.1312

[44] Murphy TF, Brauer AL, Grant BJ, Sethi S (2005) *Moraxella catarrhalis* in chronic obstructive pulmonary disease: burden of disease and immune response. Am J Respir Crit Care Med 172: 195–199. 15805178 10.1164/rccm.200412-1747OC PMC2718466

[45] Verhaegh SJ, Snippe ML, Levy F, Verbrugh HA, Jaddoe VW, et al (2011) Colonization of healthy children by *Moraxella catarrhalis* is characterized by genotype heterogeneity, virulence gene diversity and co-colonization with *Haemophilus influenzae* . Microbiology 157: 169–178. 20847012 10.1099/mic.0.042929-0

[46] Spaniol V, Troller R, Aebi C (2009) Physiologic cold shock increases adherence of *Moraxella catarrhalis* to and secretion of interleukin 8 in human upper respiratory tract epithelial cells. J Infect Dis 200: 1593–1601. 19835476 10.1086/644640

[47] Heiniger N, Troller R, Meier PS, Aebi C (2005) Cold shock response of the UspA1 outer membrane adhesin of *Moraxella catarrhalis* . Infect Immun 73: 8247–8255. 16299321 10.1128/IAI.73.12.8247-8255.2005 PMC1307079

[48] Dansky-Ullmann C, Salgaller M, Adams S, Schlom J, Greiner JW (1995) Synergistic effects of IL-6 and IFN-gamma on carcinoembryonic antigen (CEA) and HLA expression by human colorectal carcinoma cells: role for endogenous IFN-beta. Cytokine 7: 118–129. 7780031 10.1006/cyto.1995.1016

[49] Hendley JO, Hayden FG, Winther B (2005) Weekly point prevalence of *Streptococcus pneumoniae*, *Hemophilus influenzae* and *Moraxella catarrhalis* in the upper airways of normal young children: effect of respiratory illness and season. Apmis 113: 213–220. 15799766 10.1111/j.1600-0463.2005.apm1130310.x

[50] Zhang P, Chomel BB, Schau MK, Goo JS, Droz S, et al (2004) A family of variably expressed outer-membrane proteins (Vomp) mediates adhesion and autoaggregation in Bartonella quintana. Proc Natl Acad Sci U S A 101: 13630–13635. 15347808 10.1073/pnas.0405284101 PMC518805

[51] Cherry JD, Gornbein J, Heininger U, Stehr K (1998) A search for serologic correlates of immunity to *Bordetella pertussis* cough illnesses. Vaccine 16: 1901–1906. 9796041 10.1016/s0264-410x(98)00226-6

[52] Casutt-Meyer S, Renzi F, Schmaler M, Jann NJ, Amstutz M, et al (2010) Oligomeric coiled-coil adhesin YadA is a double-edged sword. PLoS One 5: e15159. 21170337 10.1371/journal.pone.0015159 PMC2999546

[53] Virji M, Evans D, Hadfield A, Grunert F, Teixeira AM, et al (1999) Critical determinants of host receptor targeting by *Neisseria meningitidis* and *Neisseria gonorrhoeae*: identification of Opa adhesiotopes on the N-domain of CD66 molecules. Mol Microbiol 34: 538–551. 10564495 10.1046/j.1365-2958.1999.01620.x

[54] Meier PS, Troller R, Heiniger N, Hays JP, van Belkum A, et al (2006) Unveiling electrotransformation of *Moraxella catarrhalis* as a process of natural transformation. FEMS Microbiol Lett 262: 72–76. 16907741 10.1111/j.1574-6968.2006.00365.x

