# Supplementary material for: A Novel Group of Moraxella catarrhalis UspA Proteins Mediates Cellular Adhesion via CEACAMs and Vitronectin
Source: PLoS One. 2012 Sep 25;7(9):e45452. doi: 10.1371/journal.pone.0045452 (PMC3458076; doi:10.1371/journal.pone.0045452)
Supplement: Figure S1 — CEACAM1 binding properties of M. catarrhalis strain 035E and its derivatives. (PDF) [file pone.0045452.s001.pdf]

Figure S1

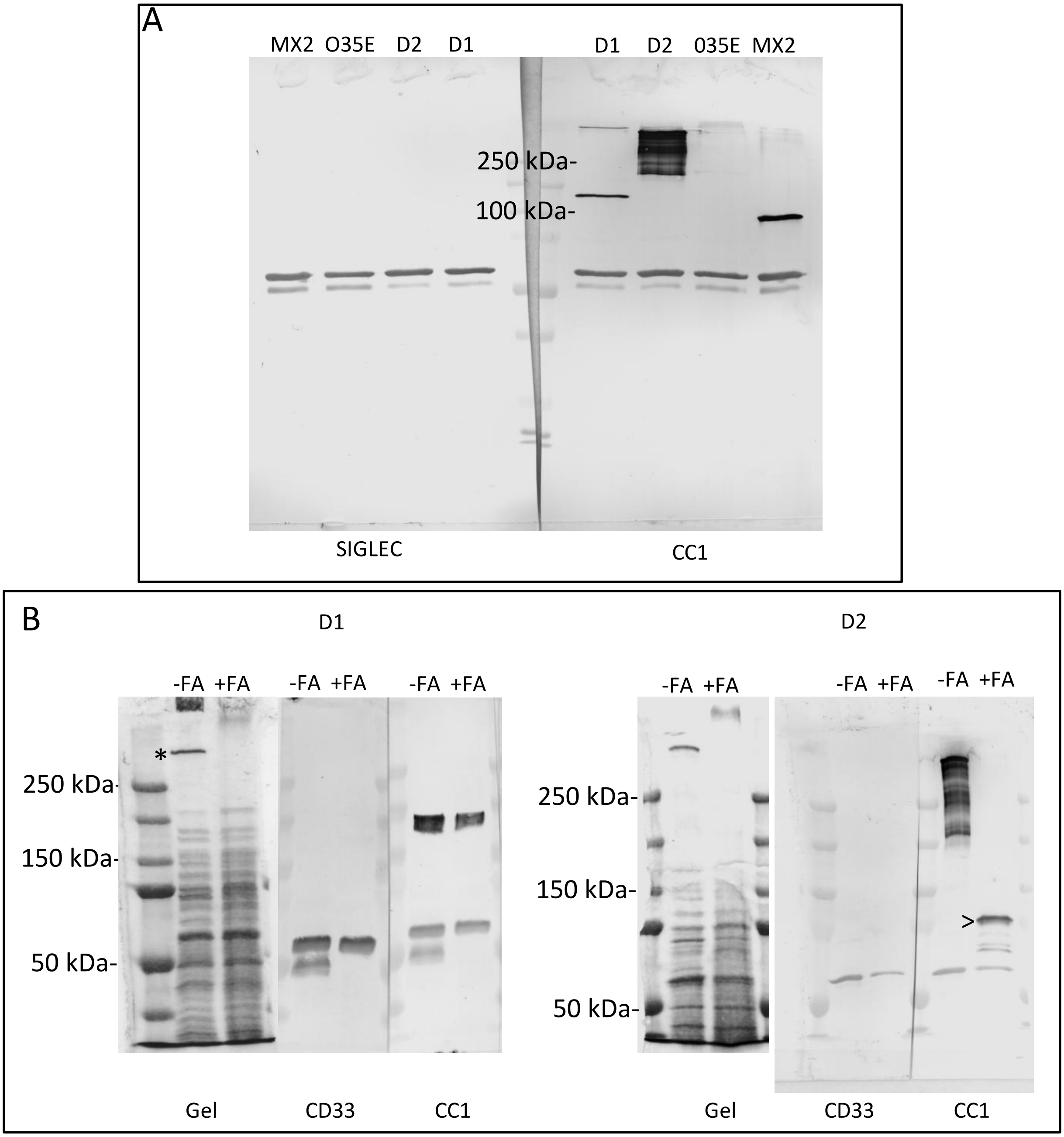

Figure S1. CEACAM1 binding properties of *M. catarrhalis* strain 035E and its derivatives. A) Western blot of Mx strains MX2 (used for comparison), 035E and 035E D2 and D1 overlaid with CEACAM1-Fc (CC1) or SIGLEC10-Fc (SIGLEC) as described in the methods. As expected, MX2 UspA1 bound to CEACAM1-Fc. No CEACAM binding protein was observed for 035E while its transformants D1 and D2 both bound to CEACAM1. Of these proteins, D2 migrated at a much higher molecular weight than expected for UspA1.

B) SDS-PAGE gels stained with Coomassie (Gel) and corresponding Western blots (CC1 and CD33) overlaid with CEACAM1-Fc and CD33-Fc respectively. Bacterial lysates of 035E D1 and D2 were preincubated without (–FA) or with 70% formic acid (+FA) and then heated at 100°C for 5 min. In the case of D1, a high molecular weight band (*) is no longer seen in the gel after formic acid treatment and one prominent CEACAM-binding band was observed in the Western blot with or without prior formic acid treatment. Thus heat alone (-FA) appears to be sufficient to induce a level of dissociation of the protein and so affect the migration of the protein, whereas formic acid treatment results in its complete dissociation (+FA). In the case of D2, formic acid treatment was required for the dissociation of the D2 high molecular weight band in the gel and correlated with the appearance of a lower molecular weight CEACAM binding band (>). Note the laddering effect on the D2 CEACAM-binding blot in the absence of formic acid is characteristic of some oligomeric coiled coil adhesins.
