# Supplementary material for: A Novel Group of Moraxella catarrhalis UspA Proteins Mediates Cellular Adhesion via CEACAMs and Vitronectin
Source: PLoS One. 2012 Sep 25;7(9):e45452. doi: 10.1371/journal.pone.0045452 (PMC3458076; doi:10.1371/journal.pone.0045452)
Supplement: Figure S2 — Novel CEACAM-binding proteins of M. catarrhalis clinical isolates. (PDF) [file pone.0045452.s002.pdf]

Figure S2

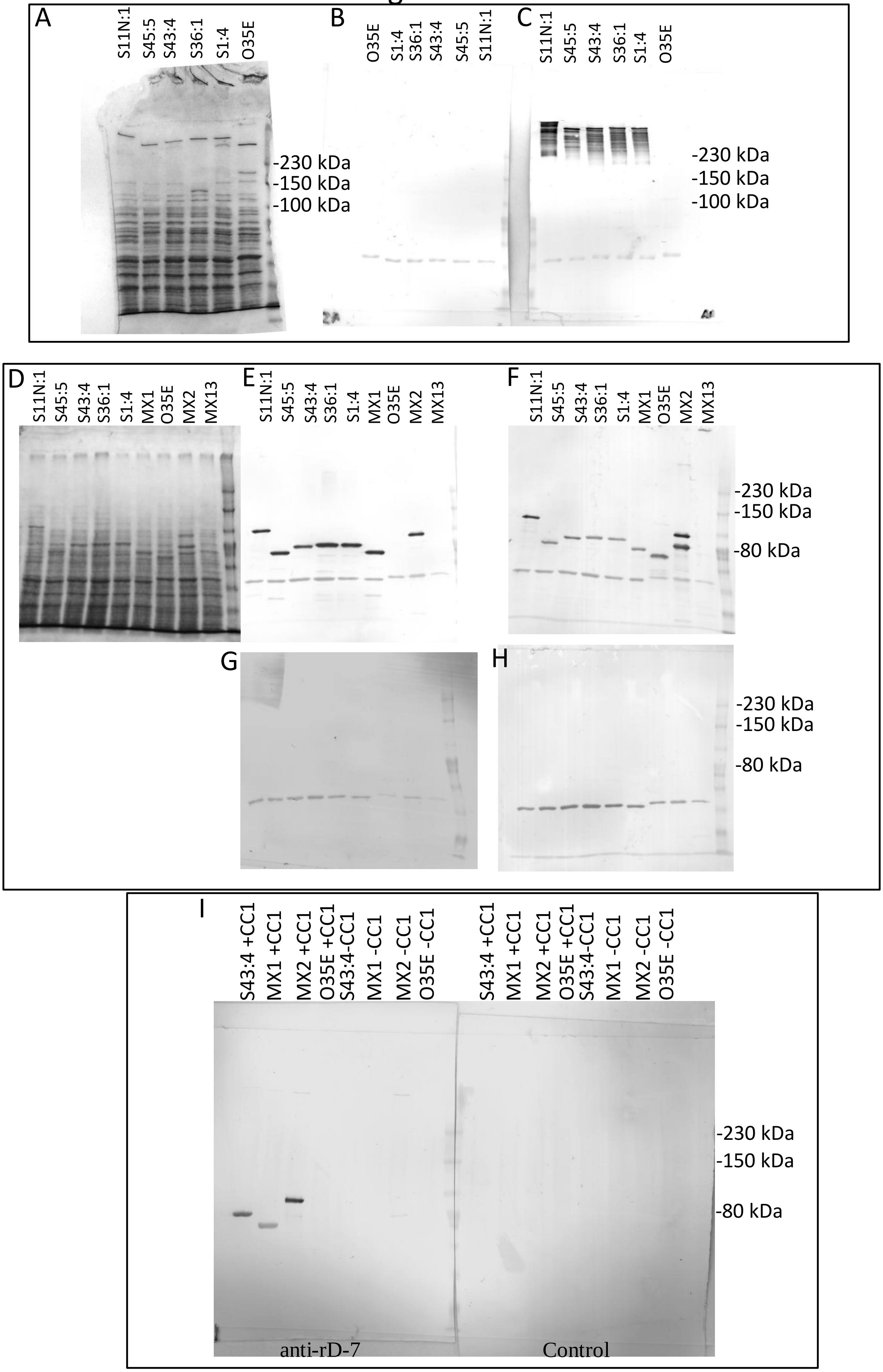

A
S11N:1
S45:5
S43:4
S36:1
S1:4
O35E
-230 kDa
-150 kDa
-100 kDa
B
O35E
S1:4
S36:1
S43:4
S45:5
S11N:1
C
S11N:1
S45:5
S43:4
S36:1
S1:4
O35E
-230 kDa
-150 kDa
-100 kDa
D
S11N:1
S45:5
S43:4
S36:1
S1:4
MX1
O35E
MX2
MX13
E
S11N:1
S45:5
S43:4
S36:1
S1:4
MX1
O35E
MX2
MX13
F
S11N:1
S45:5
S43:4
S36:1
S1:4
MX1
O35E
MX2
MX13
-230 kDa
-150 kDa
-80 kDa
G
H
-230 kDa
-150 kDa
-80 kDa
I
S43:4 +CC1
MX1 +CC1
MX2 +CC1
O35E +CC1
S43:4-CC1
MX1 -CC1
MX2 -CC1
O35E -CC1
S43:4 +CC1
MX1 +CC1
MX2 +CC1
O35E +CC1
S43:4-CC1
MX1 -CC1
MX2 -CC1
O35E -CC1
-230 kDa
-150 kDa
-80 kDa
anti-rD-7
Control

Figure S2. Novel CEACAM-binding proteins of *M. catarrhalis* clinical isolates. Representative Mx strains with novel CEACAM-binding variant proteins were subjected to SDS-PAGE and Western blotting. Gel was stained with Coomassie Blue (A) and corresponding Western blot overlaid with SIGLEC10-Fc (B) or CEACAM1-Fc (C). As observed with 035E D2, the novel CEACAM-binding proteins migrate with a higher apparent molecular weight compared to UspA1 monomers (even after heating of bacterial lysates normally sufficient to dissociate UspA1 into its monomeric form). No CEACAM binding was observed to parental 035E. (D-H) Several Mx strains were treated with formic acid prior to electrophoresis and the gel after electrophoresis was stained with Coomassie Blue (D) and the corresponding Western blots overlaid with CEACAM1-Fc (E), anti-rD-7 polyclonal antiserum (F), SIGLEC10-Fc (G) or control mouse antiserum (H). Following treatment with formic acid, the CEACAM1-binding proteins migrate with higher Mr and react with the antiserum raised against the recombinant polypeptide rD-7 encompassing the UspA1 CEACAM-binding region of Mx strain MX2. No binding of either CEACAM1-Fc or anti-rD-7 was observed to MX13 lacking expression of both UspA1 and UspA2.

(I) Western blot showing binding of anti-rD-7 antiserum (labelled) to the protein co-precipitated using CEACAM1 (+CC1) compared to the control co-precipitation which used protein A- sepharose alone (-CC1). Bands were observed at ~83kDa and 90kDa for MX1 and S43:4 respectively. UspA1 co-precipitated from MX2 migrated at ~100kDa and was detected by anti-rD-7 however, no protein detected by anti-rD-7 was co-precipitated from strain 035E. No binding was observed by the control antiserum (control).
