# Supplementary material for: A Novel Group of Moraxella catarrhalis UspA Proteins Mediates Cellular Adhesion via CEACAMs and Vitronectin
Source: PLoS One. 2012 Sep 25;7(9):e45452. doi: 10.1371/journal.pone.0045452 (PMC3458076; doi:10.1371/journal.pone.0045452)
Supplement: Figure S3 — Western Blot and PCR Analyses of the M. catarrhalis strains expressing UspA2 variant proteins not analysed further in the manuscript. (PDF) [file pone.0045452.s003.pdf]

Figure S3

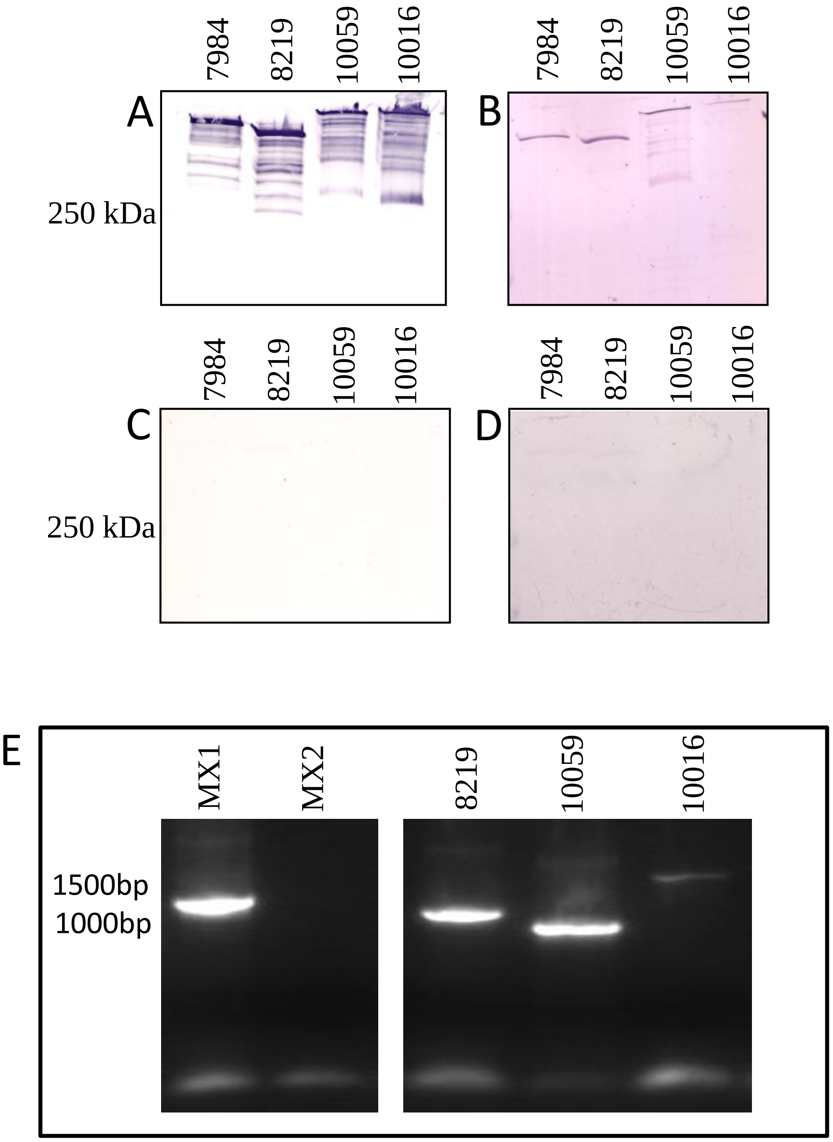

Figure S3. Western Blot and PCR Analyses of the *M. catarrhalis* strains expressing UspA2 variant proteins not analysed further in the manuscript. Strains 7984, 8219, 10059 and 10016 (as indicated) were subjected to SDS-PAGE under non-reducing conditions. Corresponding Western blots were overlaid 1ug.ml-1 CEACAM1-Fc (A) or anti-rD-7 (B). Corresponding bands were observed for CEACAM1-Fc and anti-rD-7 overlay although it should be noted that CEACAM1-Fc binding appeared to be more sensitive than anti-rD-7. No specific binding was observed with secondary antibody controls for anti-human-Fc alkaline phosphatase conjugate (C) or anti-mouse IgG alkaline phosphatase conjugate (D). E) PCR of *uspA2V* gene using *uspA2* forward primer and a reverse primer located within the CEACAM1-binding coding region of the *uspA1* gene. Note the product for stain MX1 but not MX2 which lacks *uspA2V*. In addition, products were observed for strains 8219, 10059 and 10016 as indicated.
