# Supplementary material for: A Novel Group of Moraxella catarrhalis UspA Proteins Mediates Cellular Adhesion via CEACAMs and Vitronectin
Source: PLoS One. 2012 Sep 25;7(9):e45452. doi: 10.1371/journal.pone.0045452 (PMC3458076; doi:10.1371/journal.pone.0045452)
Supplement: Figure S4 — Adhesion of the M. catarrhalis strains expressing UspA2 variant proteins to A549 human lung epithelial cells via CEACAM or vitronectin. (PDF) [file pone.0045452.s004.pdf]

Figure S4

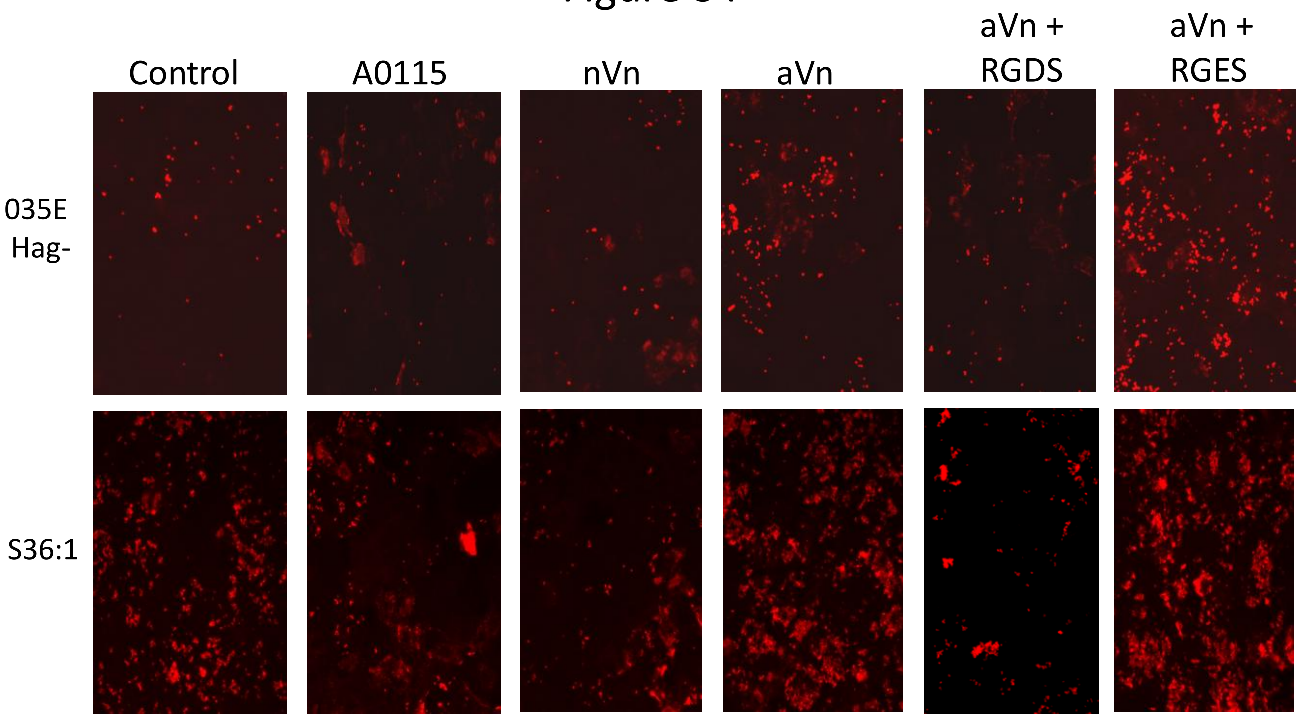

Figure S4. Adhesion of the *M. catarrhalis* strains expressing UspA2 variant proteins to A549 human lung epithelial cells via CEACAM or vitronectin. A549 cells were not treated with IFN-γ prior to infection. Cells were infected with O35E Hag- and the clinical isolate S36:1 as described in *Experimental procedures*. Infections were performed without (column 1), or with A0115 (anti-CEACAM binding polyclonal antibody; columns 2-6) in medium 199 without serum. Following infection, monolayers were fixed, blocked, and incubated with anti-Mx antisera and rhodamine conjugated secondary antibodies. A reduction in Mx binding in the presence of A0115 to S36:1 but not the non CEACAM-binding O35E parental strain can be seen from the left two columns. In addition, infections were performed in the presence of native vitronectin (nVn; column 3, activated vitronectin (aVn; column 4), aVn and RGDS (column 5) and aVn and RGES (column 6). Increased Mx binding in the presence of aVn can be seen in both cases compared with nVn. In addition, a reduction of aVn-mediated Mx binding to A549 cells was observed in the presence of RGDS but not the control tetrapeptide RGES in both cases, reflecting aVn-dependent integrin targeting observed when CEACAM interactions are prevented by A0115. Data are representative of duplicate infections performed on at least two separate occasions.
