# Supplementary material for: A Novel Group of Moraxella catarrhalis UspA Proteins Mediates Cellular Adhesion via CEACAMs and Vitronectin
Source: PLoS One. 2012 Sep 25;7(9):e45452. doi: 10.1371/journal.pone.0045452 (PMC3458076; doi:10.1371/journal.pone.0045452)
Supplement: Figure S5 — Vitronectin-mediated adherence of Mx strain S43:4 expressing the UspA2 variant protein to A549 human lung epithelial cells. (PDF) [file pone.0045452.s005.pdf]

Figure S5

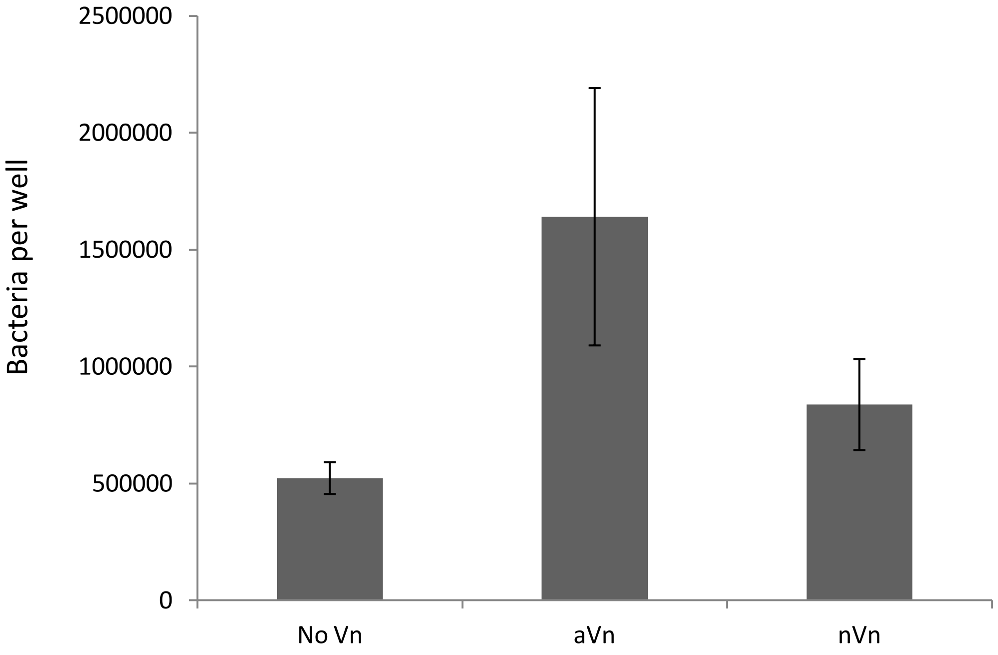

Figure S5. Vitronectin-mediated adherence of Mx strain S43:4 expressing the UspA2 variant protein to A549 human lung epithelial cells. A549 cells were pretreated with IFN-γ for 24h prior to infection in medium 199. Infections were performed in the presence of A0115 alone or supplemented with aVn or nVn as indicated. Cells were infected at an MOI of 10 for 1h. Following washing to remove non-adherent bacteria, adherent bacteria were released by the addition of saponin and appropriate dilutions plated onto HBHI agar plates. Following overnight growth at 37°C viable colonies were counted. Note the increase in adherence mediated by aVn is more apparent than that for nVn. Two independent experiments were performed each with triplicate determinations. Data are averages of means from the two experiments (n=2 +/- range).
