# Supplementary material for: A Novel Group of Moraxella catarrhalis UspA Proteins Mediates Cellular Adhesion via CEACAMs and Vitronectin
Source: PLoS One. 2012 Sep 25;7(9):e45452. doi: 10.1371/journal.pone.0045452 (PMC3458076; doi:10.1371/journal.pone.0045452)
Supplement: Figure S6 — Simultaneous CEACAM and vitronectin-mediated adherence of Mx strains expressing the UspA2 variant proteins to A549 human lung epithelial cells. (PDF) [file pone.0045452.s006.pdf]

Figure S6

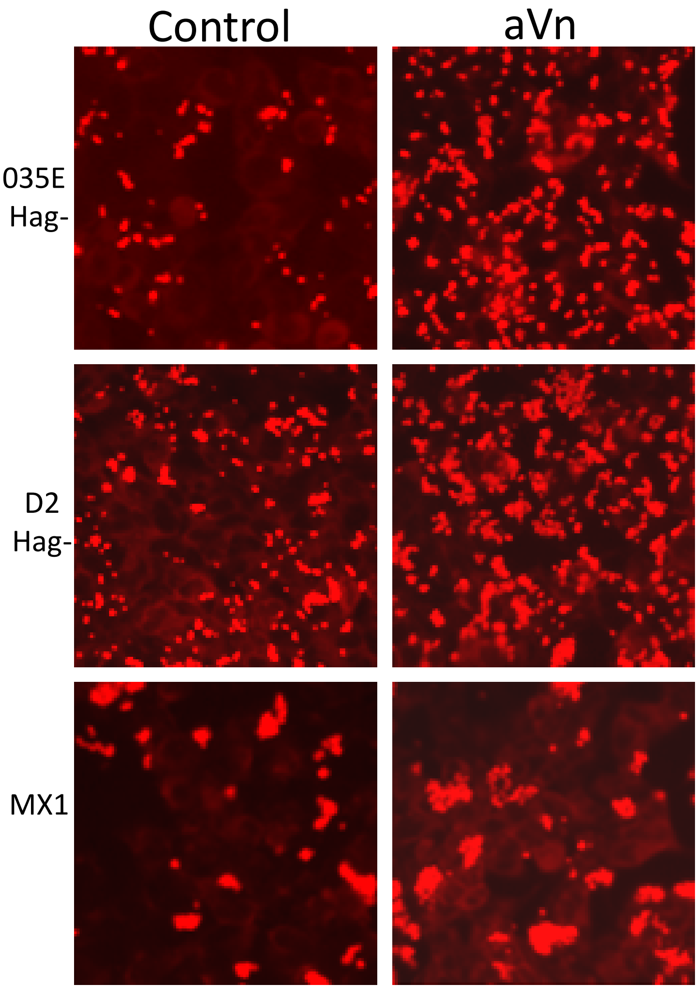

Figure S6. Simultaneous CEACAM and vitronectin-mediated adherence of Mx strains expressing the UspA2 variant proteins to A549 human lung epithelial cells. A549 cells were pretreated with IFN-γ for 24h prior to infection in medium 199 (column 1) or in medium 199 supplemented with aVn (column 2). Cells were infected with O35E Hag-, D2 and MX1 and the infected monolayers were treated as described in the legend to figure S1. Even for the CEACAM-binding derivative D2 of O35E and UspA2V containing MX1, an increase in binding is observed in the presence of aVn. In the absence of CEACAM binding of the O35E parental (Hag-) isolate, the increase-mediated by aVn is more apparent.
