# Supplementary material for: A Novel Group of Moraxella catarrhalis UspA Proteins Mediates Cellular Adhesion via CEACAMs and Vitronectin
Source: PLoS One. 2012 Sep 25;7(9):e45452. doi: 10.1371/journal.pone.0045452 (PMC3458076; doi:10.1371/journal.pone.0045452)
Supplement: Figure S7 — Binding of vitronectin directly to bacteria expressing variant UspA proteins. (PDF) [file pone.0045452.s007.pdf]

Figure S7

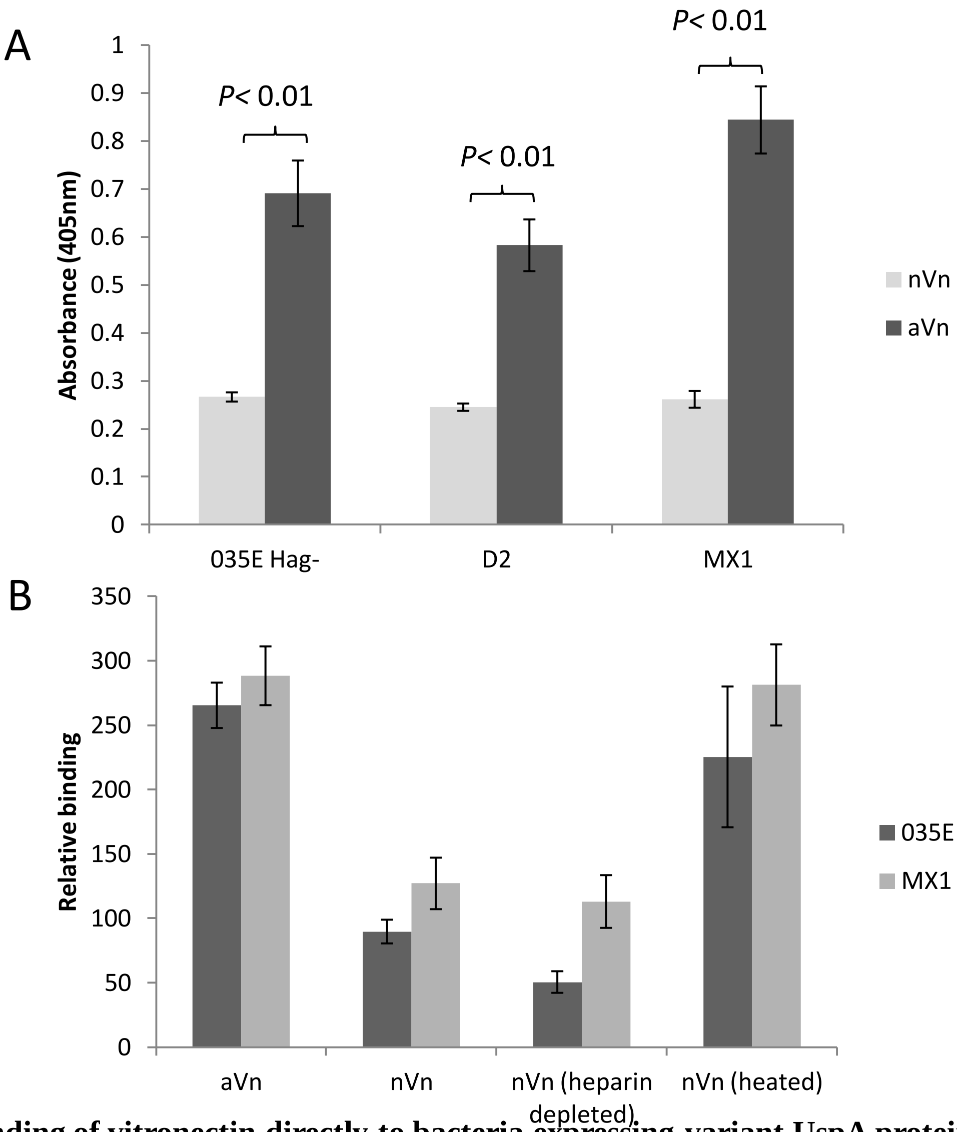

Figure S7. Binding of vitronectin directly to bacteria expressing variant UspA proteins.
A) Bacteria immobilised on to ELISA plates were overlaid with either nVn or aVn which were subsequently detected using anti-Vn polyclonal antiserum and alkaline phosphatase conjugated secondary antibody. No significant difference in activated vitronectin binding between strain 035E Hag- and D2 was observed indicating that the acquisition of CEACAM binding sequence did not interfere with vitronectin binding. Clinical strain MX1 expressing UspA2V was also able to bind aVn at similar levels to the conventional UspA2 expressed by 035E. Whilst nVn-dependent binding to epithelial cells was not observed in immunofluorescence experiments, a basal level of nVn binding was observed in the ELISA-based assay (each determination in triplicate). Data shown are overall means +/- SD from three independent experiments (n=3).
B) Bacteria immobilised on to nitrocellulose were overlaid with either nVn or aVn which were subsequently detected using anti-Vn polyclonal antiserum and appropriate secondary antibody. As observed in A, higher levels of aVn were bound to both 035E and MX1. Lower levels of nVn binding were observed which were not diminished by pre-clearance of the nVn preparation by heparin-sepharose to remove any aVn present. This low level binding could be due to partial activation of nVn during incubation with bacteria or a second lower affinity Vn-binding protein present in Mx or indeed the ability of strains to bind to nVn at low levels. To illustrate further the presence of aVn over nVn the latter preparation was subjected to heat (56°C, 15 min) to unfold the protein. Such conformation change of nVn to its activated form, results in binding levels similar to those of aVn. Data shown are means from >3 independent experiments (n=3-6) +/- SD. In each case the binding levels of nVn and aVn were statistically significantly different (*P*<0.01).
