# Supplementary material for: A Novel Group of Moraxella catarrhalis UspA Proteins Mediates Cellular Adhesion via CEACAMs and Vitronectin
Source: PLoS One. 2012 Sep 25;7(9):e45452. doi: 10.1371/journal.pone.0045452 (PMC3458076; doi:10.1371/journal.pone.0045452)
Supplement: Figure S8 — Binding of CEACAM1 and vitronectin directly to recombinant UspA2V. (PDF) [file pone.0045452.s008.pdf]

Figure S8

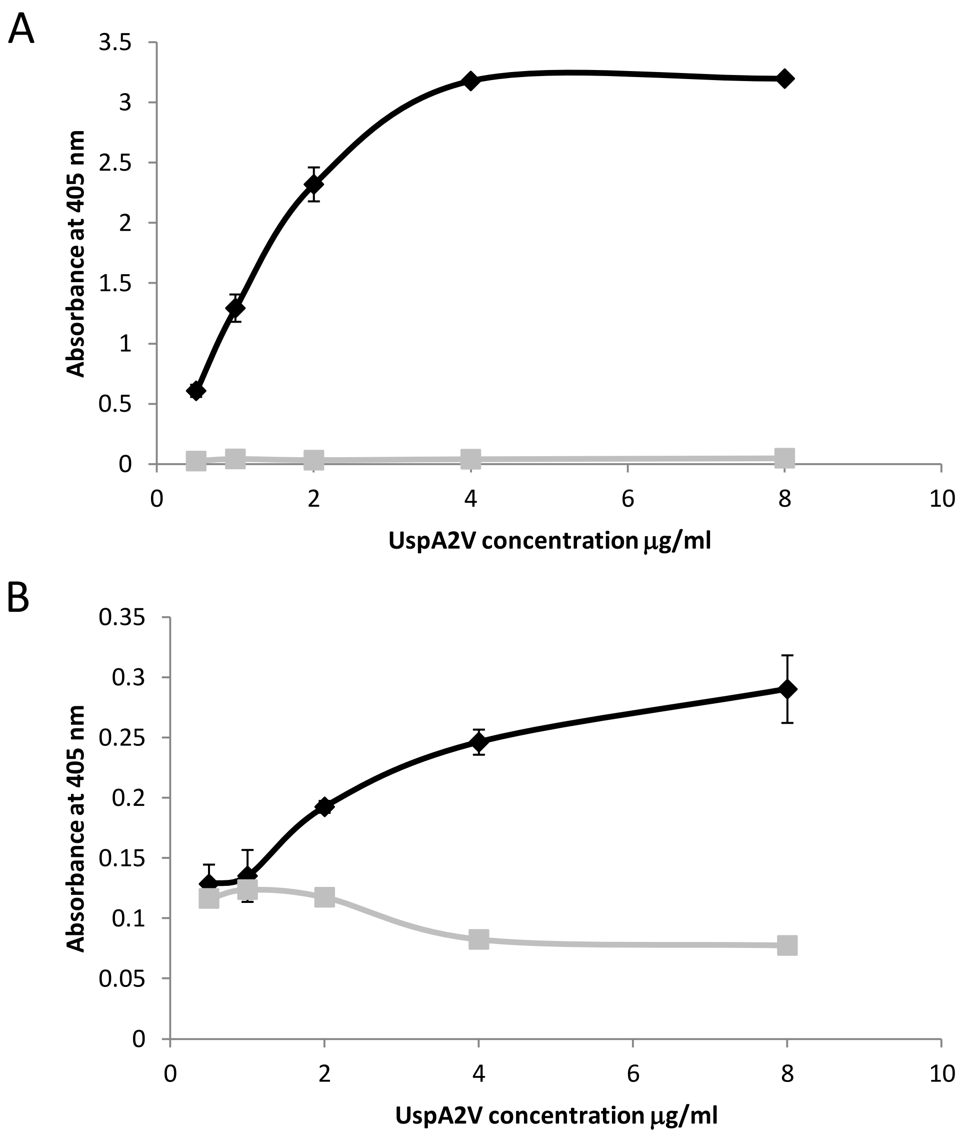

Figure S8. Binding of CEACAM1 and vitronectin directly to recombinant UspA2V.
A) Recombinant UspA2V derived from S43:4 (black diamonds) or control protein (an unrelated recombinant protein; grey squares) were immobilised on to ELISA plates at a concentration of 0.5-8 µg/ml. Immobilised proteins were overlaid with either CEACAM1-Fc (0.5 µg/ml; A) or aVn (0.5 µg/ml; B) which were subsequently detected using anti human-Fc conjugated to alkaline phosphatase or anti-Vn polyclonal antiserum and alkaline phosphatase conjugated secondary antibody respectively. Increased binding of CEACAM1-Fc was observed at all coating concentrations compared to the control protein (A). Increased binding to aVn was observed at coating concentrations above 2µg/ml compared to the control protein (B). Data shown are means +/- SD of triplicate determinations within one experiment.
